# Expression of vasopressin and its receptors in migraine-related regions in CNS and the trigeminal system: influence of sex

**DOI:** 10.1186/s10194-022-01524-7

**Published:** 2022-12-01

**Authors:** Aida Maddahi, Lars Edvinsson, Karin Warfvinge

**Affiliations:** 1 Division of Experimental Vascular Research, Department of Clinical Sciences, Lund University Hospital, Lund, Sweden; 2 Department of Clinical Experimental Research, Glostrup Research Institute, Rigshospitalet, Glostrup, Denmark

**Keywords:** Vasopressin, Oxytocin, Trigeminal ganglion, Brain, Hypothalamus, Migraine

## Abstract

**Background:**

Hypothalamus is a key region in migraine attacks. In addition, women are disproportionately affected by migraine. The calcitonin gene-related peptide (CGRP) system is an important key player in migraine pathophysiology. CGRP signaling could be a target of hormones that influence migraine. Our aim is to identify the expression of vasopressin and its receptors in the brain and in the trigeminovascular system with focus on the migraine-related regions and, furthermore, to examine the role of sex on the expression of neurohormones in the trigeminal ganglion.

**Methods:**

Rat brain and trigeminal ganglia were carefully harvested, and protein and mRNA levels were analyzed by immunohistochemistry and real-time PCR, respectively.

**Results:**

Vasopressin and its receptors immunoreactivity were found in migraine-related areas within the brain and, in the trigeminal ganglion, predominantly in neuronal cytoplasm. There were no differences in the number of positive immunoreactivity cells expression of CGRP and vasopressin in the trigeminal ganglion between male and female rats. In contrast, the number of RAMP1 (CGRP receptor), oxytocin (molecular relative to vasopressin), oxytocin receptor and vasopressin receptors (V1aR and V1bR) immunoreactive cells were higher in female compared to male rats. Vasopressin and its receptors mRNA were expressed in both hypothalamus and trigeminal ganglion; however, the vasopressin mRNA level was significantly higher in the hypothalamus.

**Conclusions:**

A better understanding of potential hormonal influences on migraine mechanisms is needed to improve treatment of female migraineurs. It is intriguing that vasopressin is an output of hypothalamic neurons that influences areas associated with migraine. Therefore, vasopressin and the closely related oxytocin might be important hypothalamic components that contribute to migraine pathophysiology.

**Supplementary Information:**

The online version contains supplementary material available at 10.1186/s10194-022-01524-7.

## Background

Migraine is the second most prevalent neurological disorder characterized as a painful, multifactorial, disabling, and recurrent neurovascular headache disorder which affects 15% of the population. Migraine involves processes that affect the brain over time. Women are disproportionately affected by migraine, representing up to 75% of all migraine cases [1–3]. The incidence of migraine attacks across the female lifespan is characteristic with incidence rises steeply at puberty, peaks during reproductive years and subside after menopause [4, 5]. However, despite the high prevalence and enormous burden of migraine among women [2, 3], the mechanisms that underlie the sex disparities in this disorder are understudied.

Although several aspects exist when it comes to the understanding of migraine pathophysiology [6], neuroimaging has played a significant role in the current understanding of processes behind migraine [7]. Positron emission tomography (PET) and functional magnetic resonance imaging (fMRI) have disclosed particular brain areas – hypothalamus and dorsal rostral pons (Pn) - that are believed to be specifically activated in migraine [8–10]. Moreover, specific nuclei in the brain stem and the midbrain, and areas of cortex, thalamus, amygdala, deep cerebellar nuclei, spinal trigeminal nuclei (Sp5) and vestibular nucleus (referred to in the text as migraine-related regions) are activated [11, 12]. It is undisputed that the dorsal pons plays a crucial role during spontaneous migraine attacks [9]. The fact that the dorsal pons shows enhanced functional connectivity with the hypothalamic region during migraine further underlines the important function of both areas for migraine pathophysiology [9]. However, much remains to be revealed regarding migraine pathophysiology. There is evidence linking the hypothalamus to the “migraine generator” in Pn, as well as with the Sp5 and sensory trigeminovascular system (TVS). This is an important way of transmission of headaches [10, 13].

Hypothalamus contains in particular two clusters of cell bodies, the paraventricular (PVN) and the supraoptic nuclei (SON), forming and storing the neuropeptides oxytocin (OT) and its molecular relative arginine vasopressin (AVP) [14]. The primary source of OT and AVP in the body are these magnocellular neurons in the hypothalamus that project to the posterior pituitary gland and secrete OT and AVP into the circulation, and with nerve fibers to different parts of the central nervous system (CNS) [15]. AVP and OT modulate nociception and pain responses by direct activation of their receptors [16, 17]. The importance of the structural characterization of the pituitary neurohormones AVP and OT was early recognized by awarding the 1955 Nobel Prize in chemistry to Vincent du Vigneaud [18]. However, we can trace back the roots of OT and AVP research to the work of G. Oliver and E. A. Schäfer, who first revealed physiological effects of the pituitary gland and its extracts in 1895 [19].The identification of structurally related hypothalamic hormones, important in regulation of blood pressure and diuresis (AVP) as well as in lactation and uterine contraction (OT), is a key advance in neuroendocrinology. AVP is considered as a stress hormone and is released in response to pain and stress, therefore having clear association with migraine pathophysiology [20].

Three G-protein coupled receptors (GPCRs) mediate the effects of AVP (V1aR, V1bR, V2R) and one single GPCR mediates the effects of OT (OTR) [21]. The OTR is richly expressed in mammary glands and uterus, the V1aR in vascular smooth muscle and the V2R in the kidneys. However, all four receptors are in addition expressed in other tissues/organs and perhaps most notably OTR, V1aR, and V1bR are widely expressed in the brain [22]. AVP and OT can be released from all parts of the magnocellular neurons i.e. also from dendrites and cell bodies [23]. OT and AVP often regulate social behaviours in sex-specific ways in adult rats [24, 25]. This may be due to sex differences in OTR and V1aR expression in the brain [25]. Neither magno- nor parvocellular AVP neurons in the PVN co-express OT. However, parvo- as well as magnocellular OT neurons express the V1b receptor, which allows them to react to locally released AVP [26]. Both OT and AVP role in facilitating social behaviours have been as evolutionary conserved as the molecular structure of the nonapeptides, their receptors, as well as the neuronal expression patterns within the brain [27–29]. Recently, we reported on OT/OTR distribution in CNS and related their expression with that of calcitonin gene-related peptide (CGRP) and regions identified to be activated in migraine [30]. CGRP is a sensory neuropeptide consistently shown to be released in excess from the trigeminal system following an acute migraine attack [31]. We found that CNS regions suggested as “migraine generators” showed correspondence to the expression of OT and OTR. Further support for their involvement in migraine was the demonstration of the ample expression of OT and OTR in the trigeminal system and their co-localization with CGRP and the CGRP receptor [30, 32]. The role of OT/OTR in modifying the CGRP system is still unknown.

We have also examined where estrogen receptors (ERα and ERβ) are expressed in the CNS and in TVS [33]. Furthermore, we specifically looked at difference in the trigeminal ganglion (TG) between males and females, both in ER expression and in the CGRP release. The most striking difference is in the amount of estrogen receptors ERα and ERβ positive cells in the female TG as compared to males.

With the background from our findings on pathways that may contribute to the role of hypothalamus in migraine attacks, the purpose of the present study is to focus attention to identify hormonal targets within the neural pathways thought to underlie migraine pathophysiology. While much data has been published regarding AVP in the brain, our focus here is on migraine-related regions.

The present study was designed to examine the expression of AVP and the AVP receptors in the CNS and TVS. Further, since migraine is a cyclic disorder, with predominance in females during their most active, reproductive years we compared with that of OT/OTR and of CGRP/CGRP receptor in male and female rats.

## Experimental procedures

### Animals

All animal procedures in this study followed the guidelines of the European Communities Council (86/609/ECC) and were approved by the Regional Ethical Committee on Animal Research, Malmö/Lund, Sweden (M17-15) and/or the Danish Animal Experimentation Inspectorate.

### Tissue preparation

A total of 12 Wistar rats were divided in 2 groups (6 males weighing approximately 250-300 g; 8-10 weeks and 6 females weighing 200-250 g; 10-12 weeks) were used for immunohistochemistry in this study. All animals were anaesthetized using CO_2_ and decapitated. Brains and TGs were carefully dissected out. The brains were cut sagittal in the midline. Thereafter, TGs and brains were fixed in 4% formaldehyde (PF) in phosphate buffer saline (PBS) for 2-4 hours at room temperature. After fixation the tissue were incubated overnight in Sörensen’s phosphate buffer (pH 7.2), gradient containing 10% and 25% sucrose (Sigma) for cryoprotection. Finally, the tissues were embedded in an egg albumin-based protein medium, cryosectioned (brain 12 μm sagittally sectioned, TG 10 μm) and stored at − 20 °C until use.

The method of PF fixation allows for a proper fixation up to 2-3 mm from the surface of the brain and inwards [34]. Since we examine a brain volume of 1 mm lateral to the midline (+ 0.5 – + 1.5 mm), a proper fixation of the tissue used for the immunohistochemistry investigation is achieved.

### Immunohistochemistry

Sections from both male and female rats were rehydrated and permeabilized in PBS containing 0.3% Triton X-100 (PBST; Sigma) for 15 min. Thereafter, the tissues were blocked for non-specific binding of antibodies for 1 hr. in blocking solution containing PBST, 1% bovine serum albumin (BSA; Sigma), and 5% normal serum. The primary antibodies were added (for details, see Table 1) and the sections were incubated in moisturized chambers at + 4 °C overnight. The following day, the sections were washed in PBST for 2 × 15 min and incubated with appropriate secondary antibodies (Table 2) for 1 hr. at room temperature. The sections were subsequently washed with PBST for 2 × 15 min and mounted with anti-fading Vectashield mounting medium containing 4′, 6-diamidino-2-phenylindole (DAPI) (Vector Laboratories, Burlingame CA, USA). Both primary and secondary antibodies were diluted in PBST and 1% BSA. The described procedure was performed in triplicate for each animal to ensure reproducibility. Further, negative controls were included by omitting the primary antibody to evaluate auto-fluorescence and non-specific secondary antibody binding levels.

**Table 1:** Primary antibodies used for immunohistochemistry

Name and product code	Dilution	Host	Supplier	References
CGRP(ab81887)	1:100	Mouse	Abcam, Cambridge, UK	Eftekhari et al. *Neuroscience*. *2010*
RAMP1(844)	1:100	Goat	Merck & Co., Inc., USA	Eftekhari et al. *Neuroscience. 2010*
OT(AB911)	1:500	Rabbit	Millipore, Temecula, CA, USA	Bean et al. *Society for Neuroscience* 2014. Jianli et al. *Comparative physiology* 2012. Hicks et al. *Neuroendocrinology 2012*
OTR(ab87312)	1:400	Goat	Abcam, Cambridge, UK	Warfvinge et al. *J Headache Pain* 2020. Krause et al *Nature Review Neurology 2021*
AVP(ab213708)	1:500	Rabbit	Abcam, Cambridge, UK	Crane-Smith Z et al. *Front Cell Dev Bio 2021*. Zhou L et al. *J Chem Neuroanat 2020.* Ran X et al. *J Chem Neuroanat 2019*
V1aR(ab187753)	1:250	Rabbit	Abcam, Cambridge, UK	Hang S et al. *Integr Zool 2021*. García-Arroyo et al. *Oxid Med Cell Longev 2020*
V1aR(bs-11598R) Cy3	1:100	Rabbit	Bioss,USA	Albee et al. *Open Biol 2018.* Yongli Shan. et al. *Neuron. 2020*
V1bR(ab104365)	1:1000	Rabbit	Abcam, Cambridge, UK	Dannenhoffer et al. *Psychopharmacology 2018*
V1bR(TA318560)	1:100	Goat	ORIGENE, Rockville, MD, USA	–
V2R(abx133128)	1:100	Rabbit	Abbexa, Cambridge, UK	Benavente MA et al. *J Comp Pathol*. *2020*
VR2(ab176488)	1:200	Rabbit	Abcam, Cambridge, UK	Ding et al. *Mol Med Rep. 2020*
Anti-CASPR (MABN69)	1:100	Mouse	Millipore, Temecula, CA, USA	Edvinsson JCA et al. *J Headache Pain. 2019*

**Table 2:** Secondary antibodies used for immunohistochemistry

Name	Dilution	Against	Supplier
Alexa 488	1:100	Goat	Thermo Scientific, IL, USA
Cy3	1:400	Goat	Jackson Immunoresearch, West Grove, PA, USA
Alxa 549	1:200	Mouse	Thermo Scientific, IL, USA
FITC	1:100	Mouse	Jackson Immunoresearch, West Grove, PA, USA
Alexa594	1:200	Rabbit	Thermo Scientific, IL, USA
FITC	1:100	Rabbit	Jackson Immunoresearch, West Grove, PA, USA

Two different antibodies for each of the AVP receptors (V1aR, V1bR and V2R) were used. Both antibodies for each receptor displayed similar results, but V1aR and V1bR from abcam and V2R from Abbexa were chosen to representatively visualize the results in the figures.

### Double immunohistochemistry

Double immunohistochemistry labeling was performed using antibodies against AVP and its receptors in combination with CGRP, RAMP1, OT, OTR or the marker of Ranvier’s nodes in myelinated axons, Contactin associated protein 1 (CASPR). The antibodies were applied and mixed as a cocktail. All procedures were the same as described above.

### Image analysis and cell counting

Immunoreactivity of the TG and the brain sections in single and double immunostainings were visualized using an epifluorescence microscope (Nikon 80i; Tokyo, Japan) at the appropriate wavelengths and photographed with an attached Nikon DS-2Mv camera. Representative images presented in the study are shown as Figs. 2, 3, 4, 5, 6, 7, 8, 9, 10, 11, 12, 13, 14, 15, 16, 17, 18 and 19.

Cell counting was performed to semi-quantify expression of AVP and its receptors, CGRP and its receptor component RAMP1 (receptor activity-modifying protein 1), OT and OTR in rat TG. Three slides (containing 3 sections)/rat, and with one slide (approx. 30 μm) between each slide used for counting, were used for measurements. Counting of cells with visible nuclei was performed in the pooled ophthalmic-maxillary and mandibular areas of each ganglion. Due to the risk of artefactual fluorescence, counting of cells close to the TG surface was not performed. Images were taken of the screen (0.75 mm^2^) at 10x magnification. A NIS-elements BR image analysis program (Nikon) was used to calculate the number of cells and to measure the fluorescence intensity in each area. All cells in this area, including negative and immunoreactive cells, were counted. The mean percentage of immunoreactive cells in 3 slides/rat (*n* = 6) was used for analyses. The intensity was measured to verify that immunoreactive cells were correctly distinguished from negative cells.

### RNA isolation and RT-qPCR

Rat hypothalamus and TG were carefully dissected out and immediately frozen on liquid nitrogen for RNA extraction from additional 6 male rats. RNA was extracted from all tissue using RNeasy® Plus Mini kit (Qiagen, Hilden, Germany) according to the manufacturer’s protocol. Total RNA concentration was determined using a GeneQuant Pro spectrophotometer (Amersham Pharmacia Biotech, Uppsala, Sweden). A ratio of sample absorbance at 260 /280 nm in the range of 1.8 to 2 was acceptable. First-strand cDNA was prepared from 1 μg of total RNA in a 20 μl reverse transcript reaction using Superscript® III First-Strand Synthesis Super Mix (Invitrogen, Carlsbad, CA, USA). A reverse transcription negative control for each sample to detect the genomic DNA was performed simultaneously and underwent the same procedures but without Superscript III Reverse Transcriptase (RT enzyme). The cDNA obtained was diluted to a total volume of 80 μL and stored at − 20 °C. The primer sequences were specific for the genes of interest and were designed using Primer Express 3.0 software (PE Applied Biosystems, Foster city, CA, USA) and synthesized by TAG Copenhagen A/S (Copenhagen, Denmark). The housekeeping gene glyceraldehyde-3-phosphate dehydrogenase (GAPDH) was used as a reference gene and the gene expressions were normalized versus that. Primers had the following sequences:

- AVP
- Forward: 5`-AGGAGAACTACCTGCCCTCG − 3`
- Reverse: 5`-AAACCCTCTCGACACTCGG-3`
- V1aR
- Forward: 5`-TCCGCACTGTGAAGATGACC-3`
- Reverse: 5`-CAACGCCGTGATTGTGATGG-3
- V1bR
- Forward: 5`-CTGGCCTACATTGCTTGCTG-3`
- Reverse: 5′-ATGGTGGCTCAAGGAACGTG-3`
- GAPDH
- Forward: 5`-CTGCACCACCAACTGCTTAGG − 3`
- Reverse: 5′-TCAGCTCTGGGATGACCTTGC- 3`

The qPCR was performed in 20 μL reaction consisting of 2 μL diluted cDNA, 0.5 μM of each primer, 10 μL Fast SYBR™ Green Master Mix (Applied Biosystems, CA, USA), and 7 μL RNAase free water in a Step One Plus Real Time PCR System (Applied Biosystems, CA, USA) with the following thermal profile: Holding stage at + 95 °C for 10 min, followed by 40 PCR cycles at + 95 °C for 15 s and + 60 °C for 1 min. Each sample was examined in duplicate and a blank control (without template) was used in all experiments. After amplification a melting curve analysis was performed to verify that each primer pair generated only one PCR product of expected size.

## Calculation and statistical analyses

Real-time PCR data were analyzed with comparative cycle threshold (C) method [35]. The C_t_ values of GAPDH, AVP, V1aR and V1bR mRNA were used as a reference to quantify the relative amount of mRNA in the sample, using the formula X_0_/R_0 =_ 2^CtR-CtX^. Where X0 is the amount of target mRNA, R_0_ is the amount of housekeeping gene mRNA, C_t_R is the C_t_ value of the housekeeping gene and C_t_X is the C_t_ value of the target.

Statistical analyses were performed using GraphPad Prism. Statistical significance was determined using Kruskal Wallis non-parametric test with Dunn’s post-hoc test. Data were expressed as mean ± standard error of the mean (s.e.m.), n refers to the number of rats, *P < 0.05, **P < 0.01 and ***P < 0.00 were considered significant.

## Results

### Immunohistochemistry

Previous studies have demonstrated distribution of CGRP, RAMP1, OT, OTR, and estrogen receptors ERα, ERβ and GPER in the TGs of young male rat [32, 33]. To examine possible differences in expression of these proteins in male and in female rats, we used indirect immunofluorescence staining for detection and localization of these proteins. Furthermore, the distribution of AVP and AVP receptors (V1aR, V1bR) in TG and in CNS were scrutinized.

### Distribution of vasopressin and vasopressin receptors in the male rat brain

**Fig. 1:**
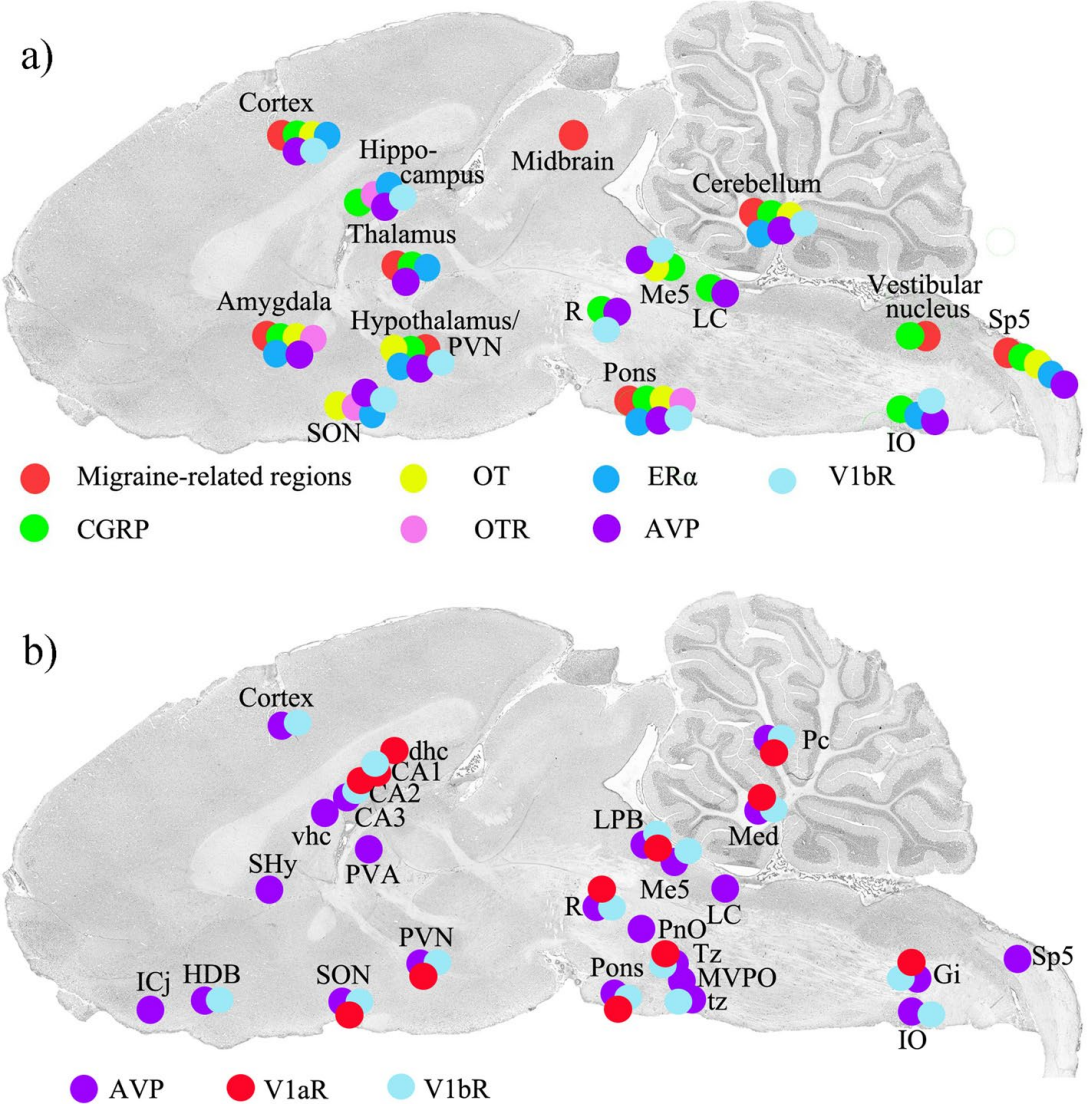
**a** Comparison of CGRP, OT/OTR, ERα, AVP and V1bR with migraine-related regions. **b** Distribution of AVP and its receptors

We have compared brain areas identified in imaging studies of humans experiencing a migraine attack [36] with the distribution of CGRP, OT, AVP, and their individual receptors, and estrogen receptors (Fig. 1a). The results of our AVP mapping study of the brain are summarized in Fig. 1b.

### Son/PVN

**Fig. 2:**
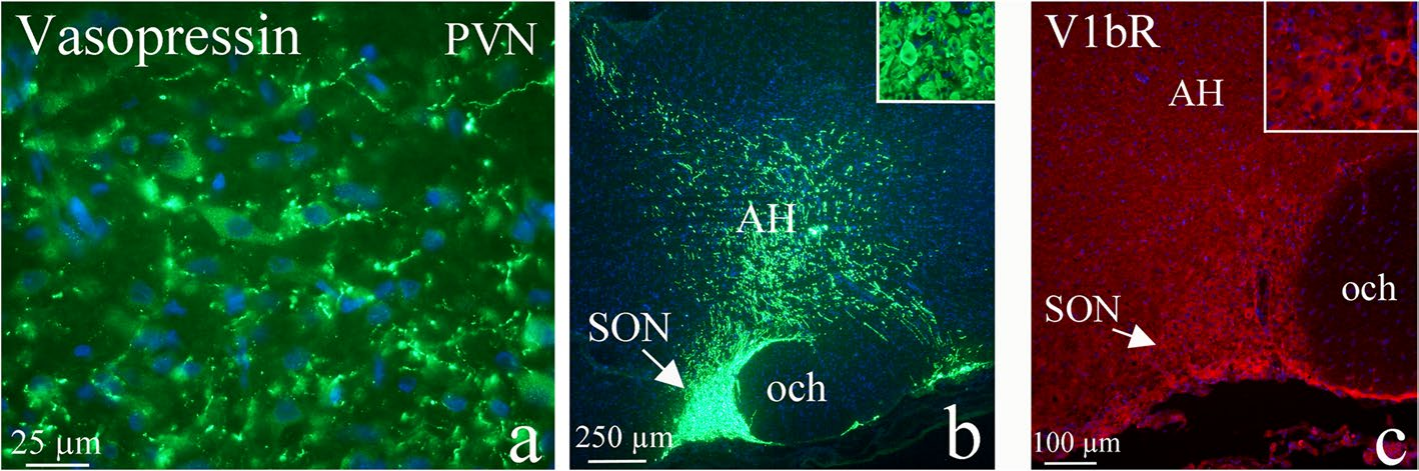
AVP and its receptors in PVN and SON. **a** AVP was expressed in numerous neuronal soma within PVN. **b** Neurons within SON showed intense expression of AVP. Arrow points at the intense immunoreactivity in SON. In addition, fibers expressing AVP were spreading within AH. **c** Positive V1bR neurons were found in SON (arrow) and in AH. However, AVP immunoreactivity was more apparent than V1bR

As expected, AVP was expressed in numerous neuronal soma within the supraoptic (SON) and paraventricular (PVN) nuclei; especially neurons within SON showed intense expression of AVP, however, the expression of AVP was less intense in the PVN (Fig. 2a and b). In addition, immunoreactive fibers were found within both nuclei, but were also spreading outside the nuclei. It was more apparent for SON originating fibers, which spread through the anterior hypothalamic area (AH), a heterogeneous collection of cells. It is known that projections of hypothalamic neurons in PVN and SON nuclei are widespread throughout the brain and spinal cord [37–39]. Both V1aR and V1bR expression were observed in SON/PVN (Fig. 2c).

### Cerebral cortex, cerebellum and medial cerebellar nucleus

**Fig. 3:**
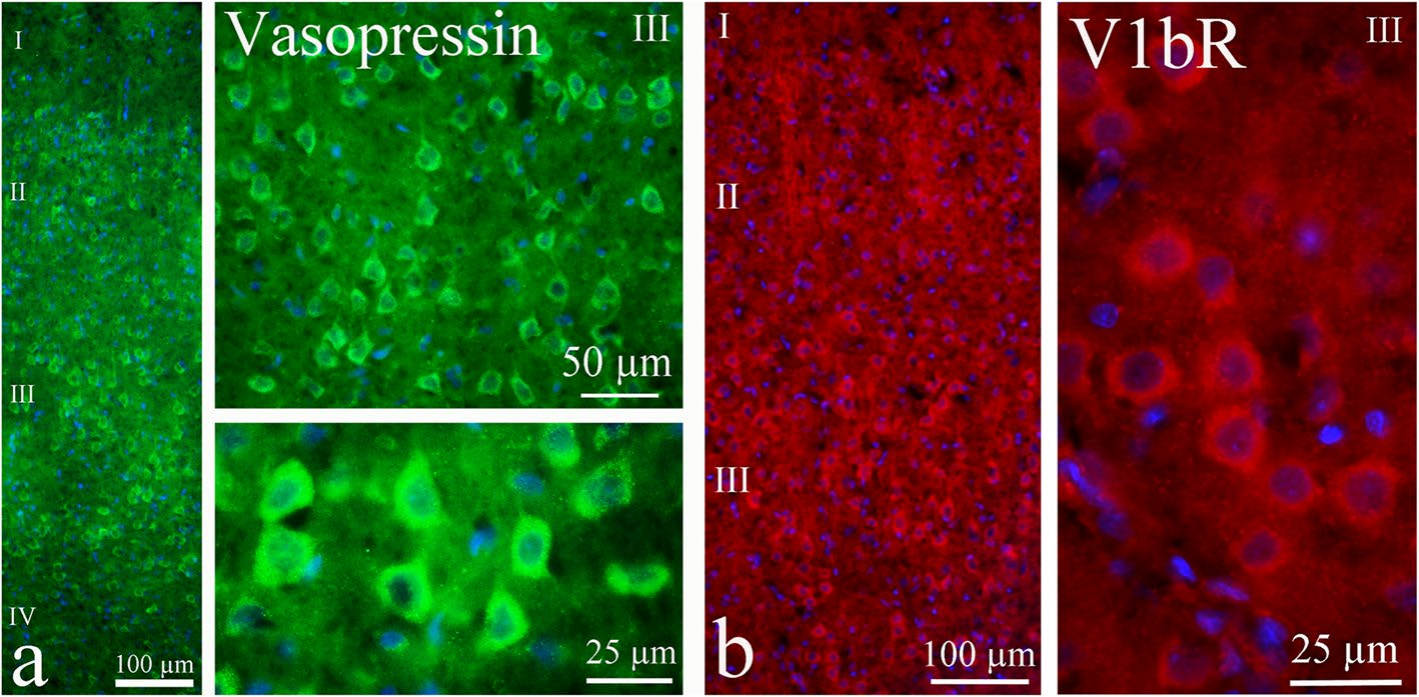
AVP and its receptors in the cerebral cortex. **a** AVP expression was mainly found in layers II and III. The expression was observed in the cell soma, often visualized as positive grains in the cytoplasm. The neuronal processes showed very limited or no immunoreactivity. No AVP immunoreactivity was detected in cortical layer I, where there were few neuronal cell somas. **b** V1bR immunoreactivity was observed mainly in layer III. The expression was displayed in the neuronal cytoplasm. No staining was found in the fibers

In cerebral cortex, especially in the occipital cortex, AVP expression was mainly found in layers II and III (Fig. 3a). The expression was observed in the cell soma, often visualized as positive grains in the cytoplasm in the neurons. The neuronal processes showed very limited or no AVP immunoreactivity. However, pyramidal neurons could be seen to express AVP in the efferent fibers, which target other areas within the brain. Occasional neurons in layers IV-VI showed AVP immunoreactivity. Region close to cerebral cortex - the white matter of corpus callosum (cc) - which connects the two hemispheres, did not display AVP immunoreactivity. In addition, cortical layer I, which harbours few neuronal cell soma, did not show AVP immunoreactivity.

We did not observe any V1aR expression in the cerebral cortex. V1bR immunoreactivity was however observed mainly in layer III (Fig. 3b). The expression was displayed in the neuronal cytoplasm; there was no staining of the fibers.

**Fig. 4:**
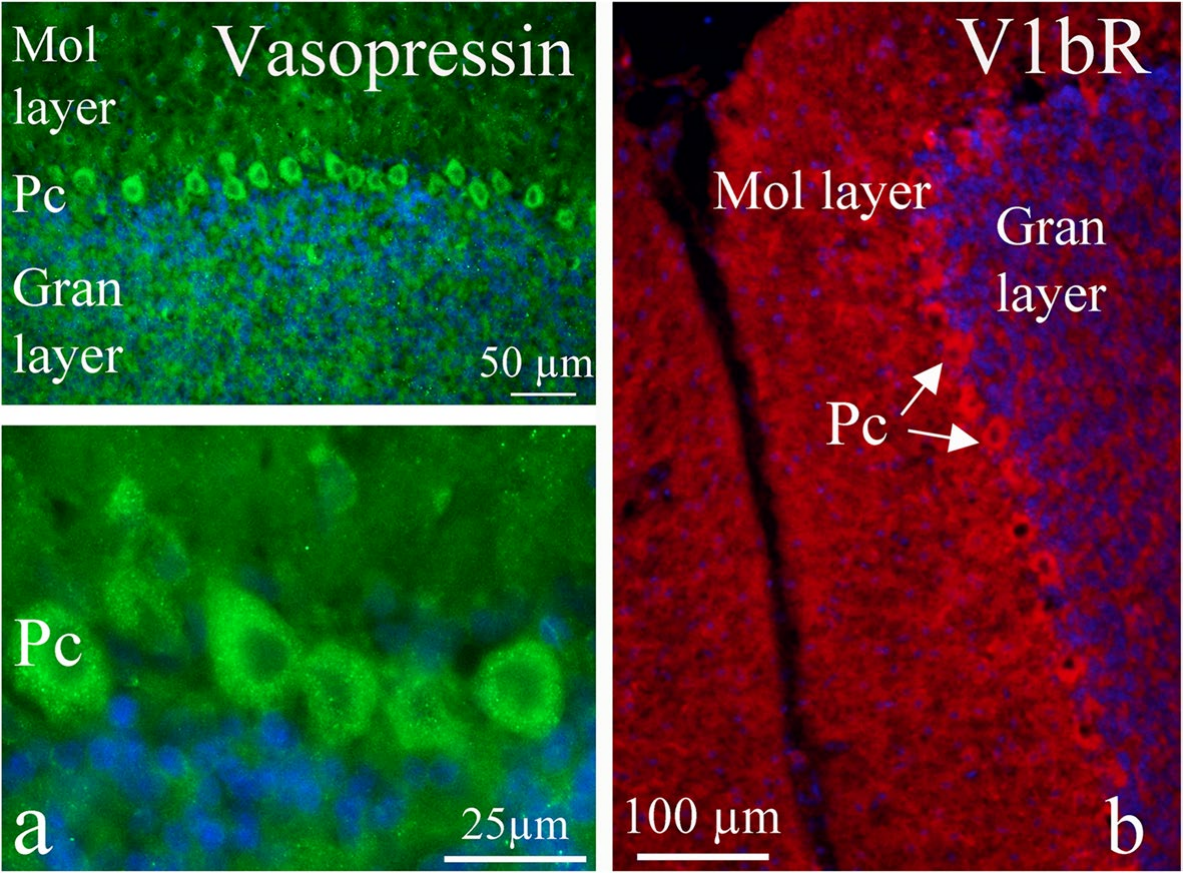
AVP and its receptors in the cerebellum. **a** AVP expression was mainly found in the Purkinje cell (Pc) soma. The expression was visualized as positive grains in the cytoplasm, as was seen in the cerebral cortical neurons. **b** V1bR immunoreactivity was found in both molecular, Pc (arrows) and granular cell layers

In the cerebellum, AVP expression was mainly found in the Purkinje cell (Pc) soma (Fig. 4a). The expression was visualized as positive grains in the cytoplasm, as was seen in the cerebral cortical neurons. Both V1aR and V1bR were expressed in cerebellum, however, V1aR expression was mainly found in the Pc, with weak staining intensity. V1bR immunoreactivity was found in both molecular, Pc and granular cell layers (Fig. 4b).

**Fig. 5:**
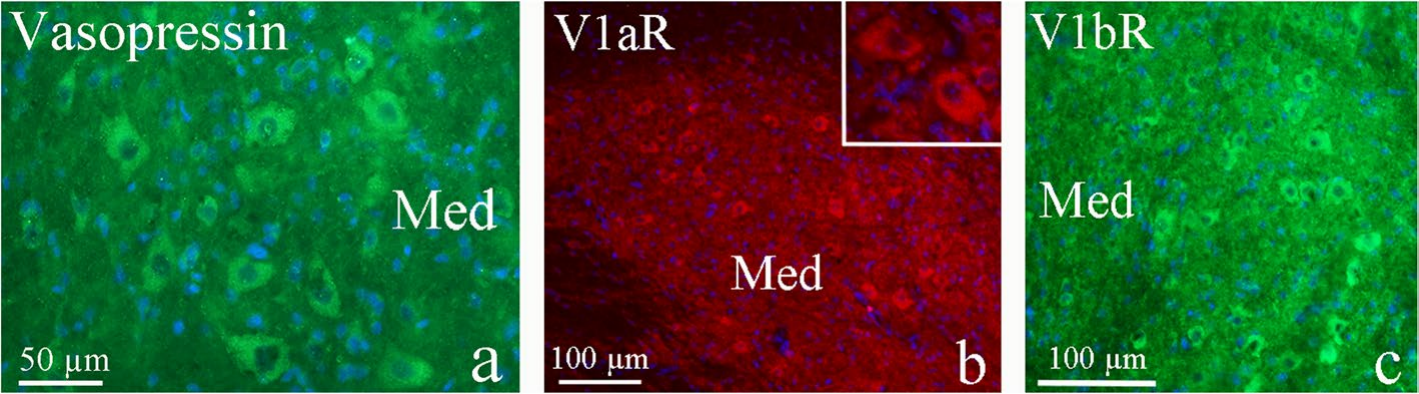
AVP and its receptors in the medial cerebellar nucleus (Med). The large neurons of Med showed AVP (**a**), V1aR (**b**) and V1bR (**c**) immunoreactivity in the neuronal cytoplasm

The medial cerebellar nucleus (Med) is one of the four nuclei embedded in the white matter of the cerebellum. Most output fibers from the cerebellum originate from these nuclei. The input originates from Pc and hippocampal mossy fibers. The large neurons of Med showed AVP, V1aR and V1bR immunoreactivity in the neuronal cytoplasm (Fig. 5a-c).

### Hippocampus

The hippocampus is an enfolding of the cerebral cortex with a shape in cross-section of a sea horse. It consists of grey matter which is covered with white matter. The hippocampus is divided into CA1, CA2, CA3 (which process, send and receive input from different brain regions) and the dentate gyrus. These areas are functionally and anatomically distinct.

**Fig. 6:**
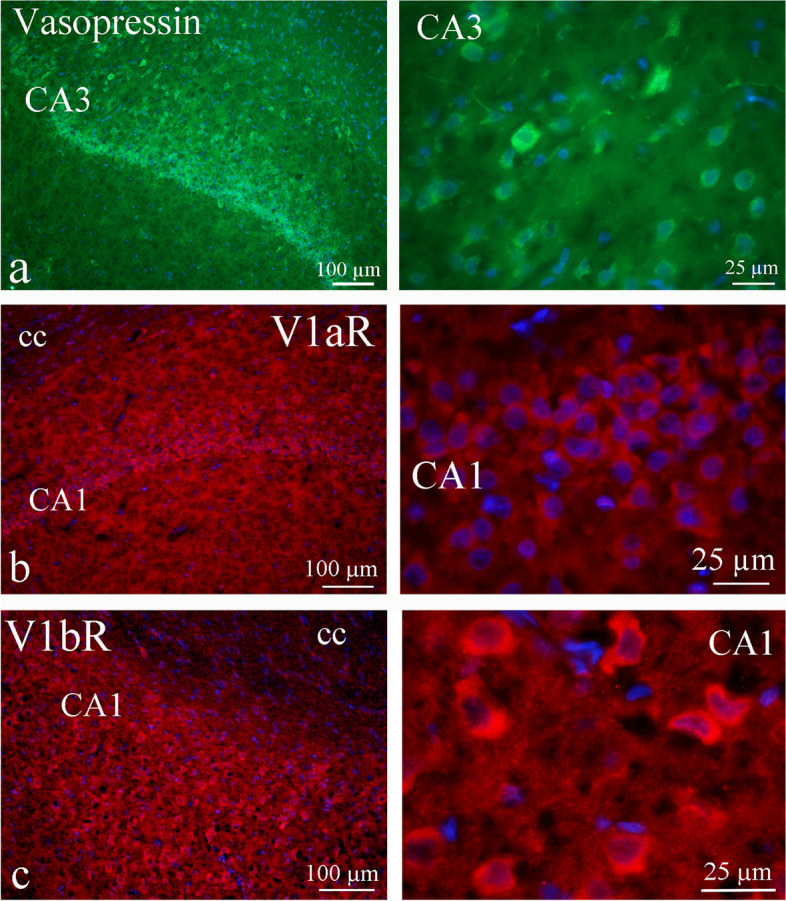
AVP and its receptors in the hippocampus. **a** AVP expression was found in the hippocampus area CA3. The different neurons in CA3 were AVP immunoreactive; however the cells displayed different immunoreactive intensity (right column). The pyramidal cell displayed more intensity than the smaller neurons. In addition, some fibers expressed AVP. **b** and **c** The receptors were expressed in a similar manner in the CA1 hippocampal region and in the neuronal cytoplasm

We observed AVP expression in the hippocampus area CA3. The different neurons in CA3 were AVP immunoreactive; however, cells displayed different immunoreactive intensity, as can be seen in the right column of Fig. 6a.

**Fig. 7:**
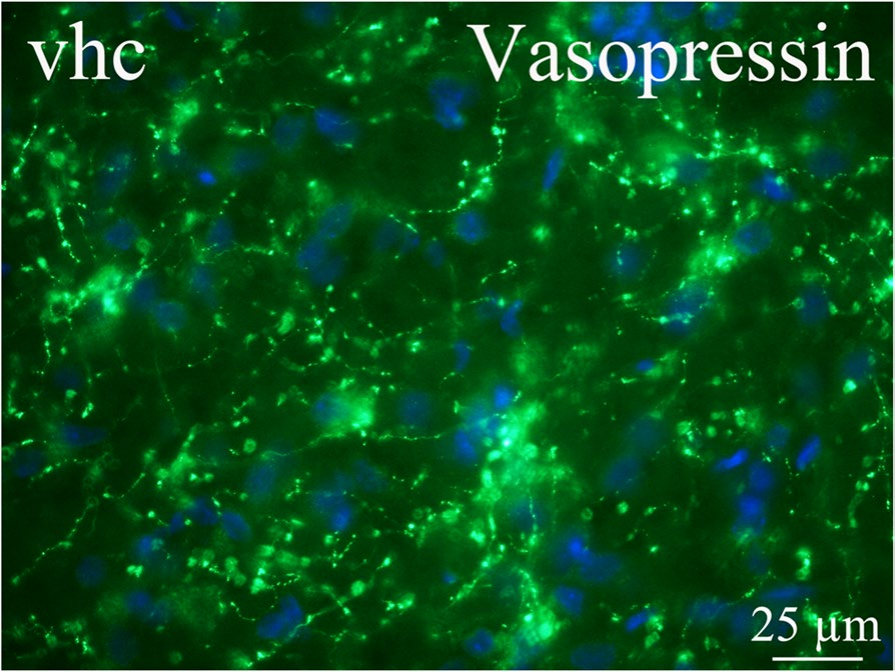
AVP in the ventral hippocampal commissure (vhc). The fiber region closely related to hippocampus, the ventral hippocampal commissure (vhc), showed vasopressin expression in the fibers in a pearl-like pattern

The pyramidal cells displayed higher intensity than the smaller neurons. In addition, some fibers expressed AVP. The closely related fiber region – the ventral hippocampal commissure (vhc) – showed AVP expression in the fibers in a pearl-like pattern (Fig. 7).

The AVP receptors were expressed in a similar manner; in the CA1, CA2 and CA3 hippocampal regions and in the neuronal cytoplasm. In addition, V1aR immunoreactivity was observed in the dorsal hippocampal commissure (dhc). Still, the V1aR expression was weaker (Fig. 6b) than the V1bR expression (Fig. 6c).

### Thalamus and hypothalamus

The thalamus is one of the most complex structures in the brain and is involved in nearly all of its activities. Much work has indicated that the thalamus and the hypothalamus are possible sites of origin for the migraine attack [36]. AVP, V1aR and V1bR expression were found in the paraventricular thalamic nucleus (PVA). The AVP immunoreactivity in the hypothalamus was mainly, as described above, found in and around PVN and SON (Fig. 2). However, in the septohypothalamic nuleus (SHy) some pearl-like fibers expressed AVP.

### Brain stem

**Fig. 8:**
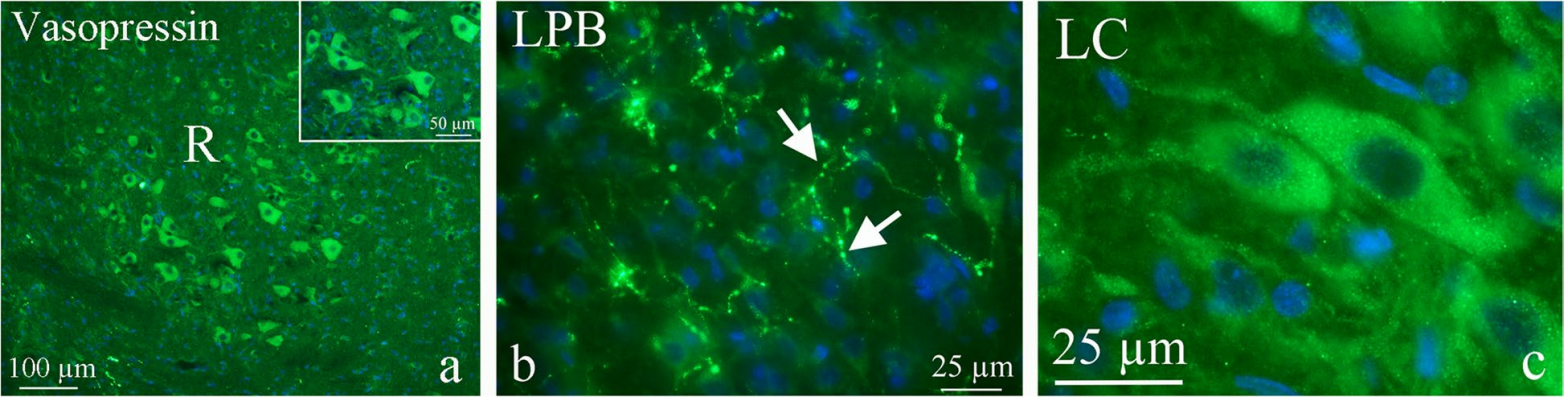
AVP in the red nucleus (R), lateral parabrachial nucleus (LPB) and locus coeruleus (LC). **a** Large neurons in R showed intense AVP immunoreactivity and in addition, some positive fibers were found in this region. **b** LPB showed AVP expression in the fibers in a pearl-like pattern (arrows) and **c** in the LC large bipolar neurons displayed granular staining in the cytoplasm

In the brain stem, some nuclei displayed AVP and AVP receptor expression such as the red nucleus (R; involved in the motor coordination, and part of the pathway that influence cerebellum), lateral parabrachial nucleus (LPB; receive information from a variety of sources in the brainstem, transmit signals to hypothalamus), mesencephalic trigeminal nucleus (Me5; contain cell bodies of first order sensory neurons, involved with reflex proprioception of the face) and locus coeruleus (LC; involved with responses to stress and panic, project far and wide). Large neurons in R showed intense AVP immunoreactivity and in addition, some positive fibers were found in this region (Fig. 8a). As for vhc, LPB showed AVP expression in the fibers (presumably C-fibers) in a pearl-like pattern (Fig. 8b). The AVP expression in Me5 was weaker than the other nuclei in this area. In LC, large bipolar neurons displayed granular staining in the cytoplasm (Fig. 8c).

V1aR expression was only found in LPB and R. On the other hand, V1bR immunoreactivity was displayed in R, LPB and Me5. However, the intensity of the immunoreaction was weaker than the AVP expression for all receptors staining in the brainstem area.

### Region of the pons

**Fig. 9:**
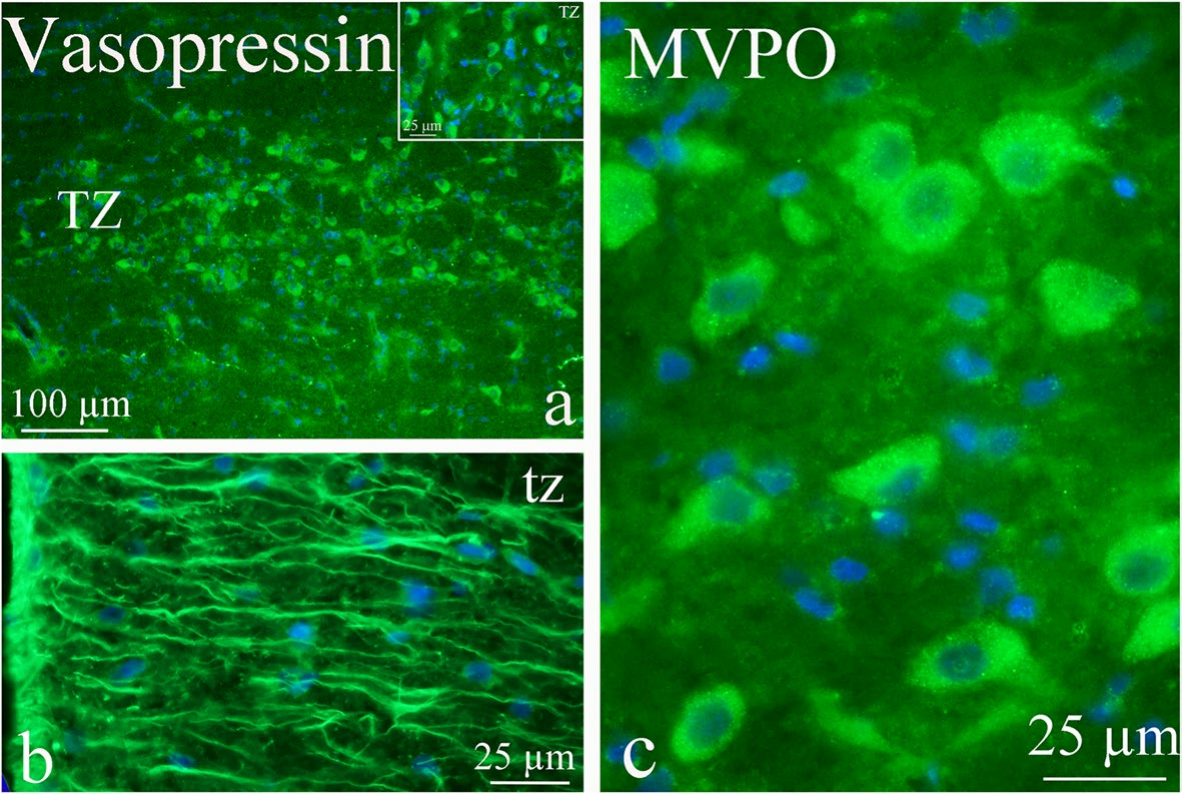
AVP in the nucleus of trapezoid body (Tz), trapezoid body (tz) and medioventral peri olivary nucleus (MVPO). **a** AVP expression was observed in **a** the nucleus of trapezoid body (Tz) and **b** trapezoid body (tz), and **c** medioventral periolivary nucleus (MVPO). Few immunoreactive fibers were found except for Tz

Pontine nuclei are important brainstem areas for trigeminal nociceptive processing and pain pathology [12]. AVP expression was observed in the pontine nuclei (Pn), pontine reticular nucleus (PnO), nucleus of trapezoid body (Tz) and trapezoid body (tz), and medioventral periolivary nucleus (MVPO) (Fig. 9a-c). The neurons showed intense immunoreactivity in the cytoplasm in the nuclei of the pontine area (Fig. 9c). Few immunoreactive fibers were found except for Tz that showed intense fiber staining (Fig. 9b).

**Fig. 10:**
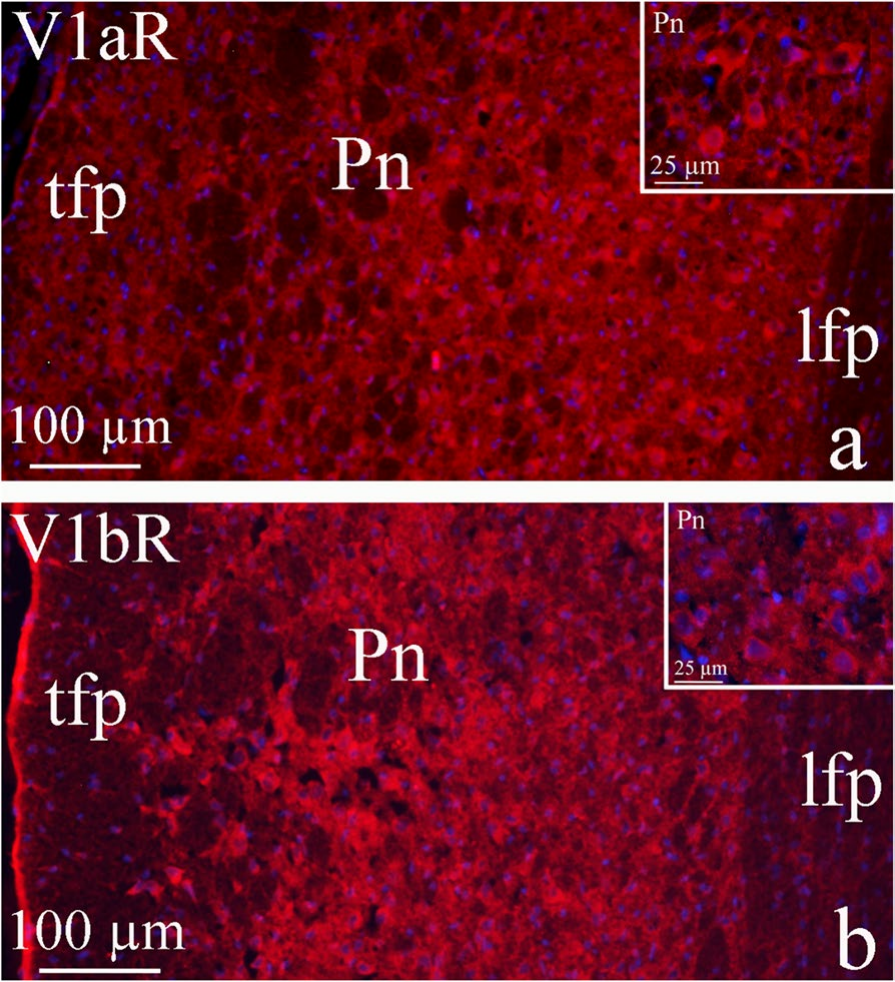
AVP receptors in the pontine nuclei (Pn).V1aR and V1bR were expressed in a similar matter in Pn except for transverse fibers pons (tfp) (Fig. 10a and b). **a** The V1aR immunoreactivity showed an almost background like pattern, yet, neurons within Pn showed intense immunoreactivity in the neuronal cytoplasm. **b** The neurons expressed intense immunoreactivity in the cytoplasm, as did in V1aR immunohistochemistry. V1aR and V1bR were expressed in a similar matter in Pn except for transverse fibers pons (tfp) (arrows) that were observed in V1aR staining (**a**), but not in V1bR (**b**). The longitudinal fasciculus of the pons (lfp) did not show any expression of the two receptors

V1aR and V1bR were expressed in a similar matter in Pn except for transverse fibers pons (tfp) (Fig. 10a and b). The V1aR immunoreactivity showed an almost background like pattern (Fig. 10a), yet, neurons within Pn showed intense immunoreactivity in the neuronal cytoplasm as displayed with V1bR immunohistochemistry.

### Medulla oblongata

**Fig. 11:**
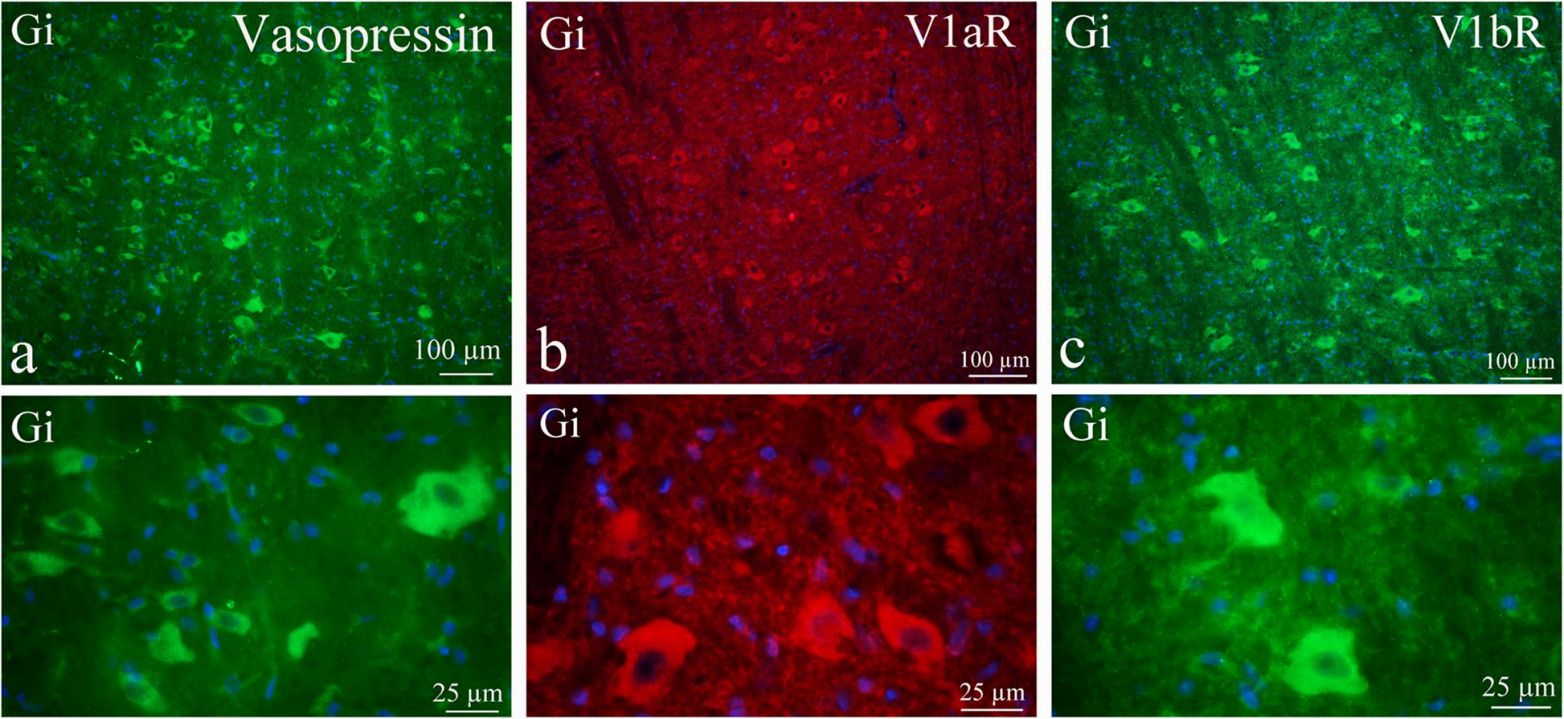
AVP and its receptors in the Gigantocellular reticular nucleus (Gi). **a** These large neurons expressed AVP in their cytoplasm, but also V1aR (**b**) and V1bR (**c**). No difference could be seen between AVP and its receptor in the distribution or expressional intensity

As the name of the nucleus suggests - Gigantocellular reticular nucleus (Gi) – the neurons are large. This nucleus has been known to innervate the hypoglossal nucleus. These cells expressed AVP in their cytoplasm, but also V1aR and V1bR (Fig. 11a-c). No difference could be seen between AVP and its receptor in the distribution or expressional intensity.

**Fig. 12:**
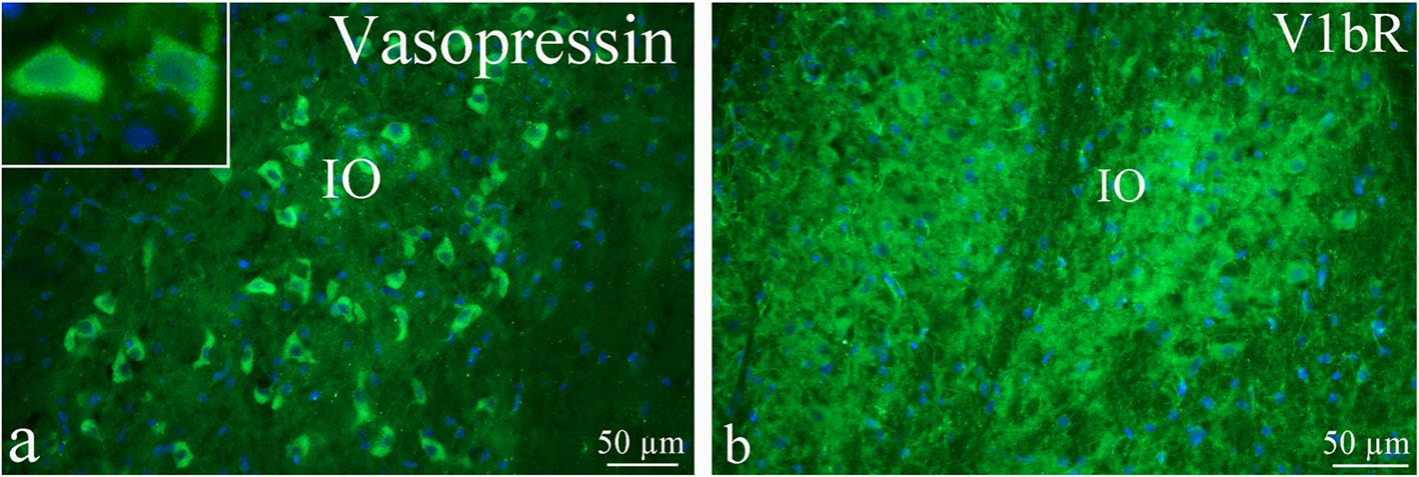
AVP and its receptors in the Inferior olive (IO). **a** The AVP expression of the inferior olive was found in the neuronal cytoplasm, but not in the fibers. **b** V1bR expression was observed in the neuronal cytoplasm, but also in the surrounding fibers

The AVP expression of the inferior olive (IO) was found in the neuronal cytoplasm, but not in the fibers (Fig. 12a). IO is known to coordinate signals from the spinal cord to the cerebellum to regulate memory and learning. V1bR expression was observed in the neuronal cytoplasm, but also in the surrounding fibers (Fig. 12b).

No V1aR immunoreactivity was found in IO. In Sp5 which receives trigeminal pain signals originating in the meninges, some fibers displayed AVP immunoreactivity, but not the receptors.

### Islands of Calleja

**Fig. 13:**
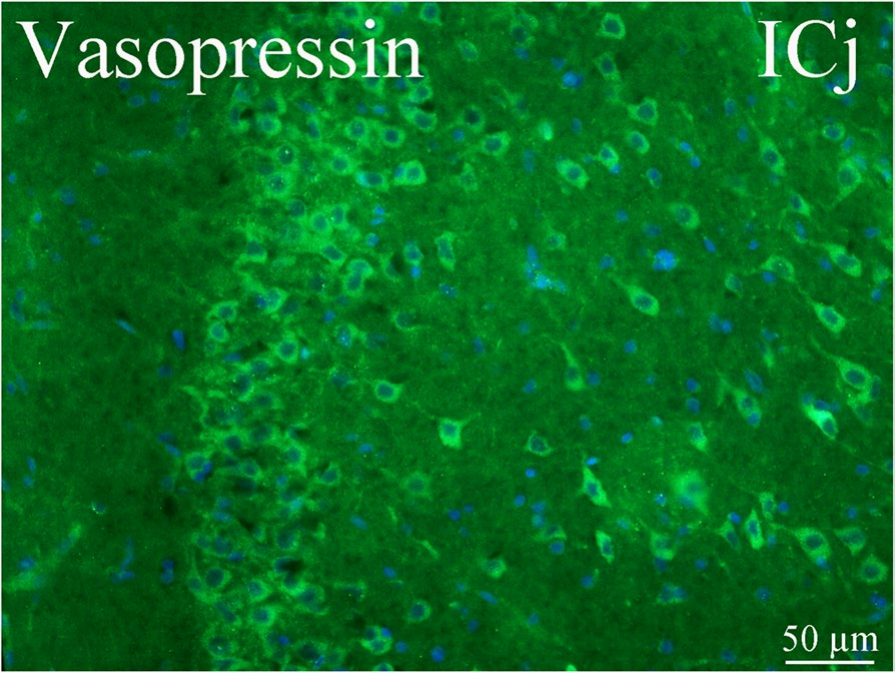
AVP and its receptors in the Islands of Calleja (ICj). We found AVP immunoreactive neurons in this area

The islands of Calleja (ICj) are a group of neuronal granule cells that are located within the olfactory tubercle in rodents. This region is part of the limbic system, where it aids in the reinforcing effects of reward-like activities. Anatomical studies have shown that the amygdala gives rise to important projections to the olfactory tubercle and the Islands of Calleja, suggesting that these amygdala-striatal pathways might be involved in the reinforcing value of sexual pheromones [40]. We found AVP immunoreactive neurons in this area (Fig. 13).

### Comparison of expression of CGRP, oxytocin, estrogen, vasopressin and their receptors in the trigeminal ganglion of male and female rats

#### Distribution of CGRP and RAMP1 in the trigeminal ganglion

**Fig. 14:**
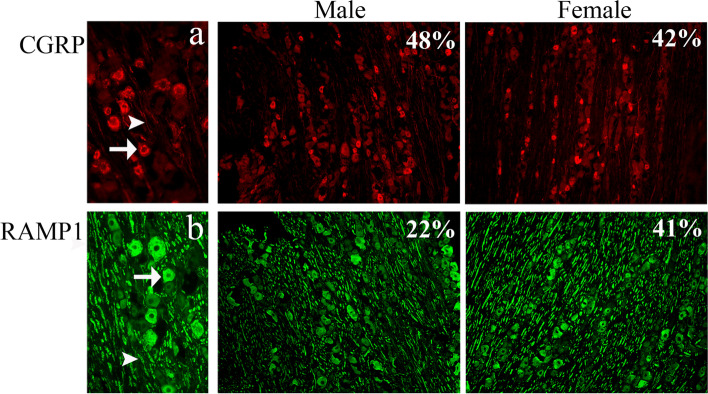
Immunohistochemistry of CGRP and RAMP1 expression in TG from male and female rat. **a** CGRP was located in the cytoplasm of neurons (arrow) and in C-fibers (arrowhead). 48% in male and 42% in female of the total number of neurons showed positive CGRP immunoreactivity. **b** 22% in males and 41% in females of all neurons was expressed RAMP1 which was located in the neuronal cytoplasm (arrows) and in thick nerve fiber (Aδ –fibers) (arrowheads)

We calculated the number of CGRP expressing neurons and found that 48% of the total number of neurons in males and 42% in females expressed CGRP immunoreactivity in TG, which is in agreement with an earlier study from our laboratory by Eftekhari et al. on human TG [41]. In the present study as well as in our earlier study, the CGRP immunoreactivity was detected mostly in the cytoplasm in a granular-like pattern of small and medium sized neurons. Moreover, we found CGRP immunoreactivity in thin fibers (presumably C-fibers) (Fig. 14a). There was no difference in the number of CGRP immunoreactive cells between male and female rats (Table 3).

**Table 3:** Cell counting in rat trigeminal ganglion

	CGRP	RAMP1	OT	OTR	AVP	V1aR	V1bR
**N**	Male Female	Male Female	Male Female	Male Female	Male Female	Male Female	Male Female
1	52% 44%	14% 44%	33% 48%	40% 30%	34% 39%	65% 88%	84% 82%
2	46% 37%	25% 43%	41% 56%	34% 37%	35% 44%	85% 86%	69% 87%
3	45% 42%	29% 36%	32% 50%	24% 52%	41% 36%	80% 88%	84% 85%
4	53% 35%	20% 47%	35% 51%	35% 58%	33% 30%	73% 85%	78% 82%
5	50% 48%	22% 46%	40% 65%	30% 50%	34% 38%	83% 77%	66% 79%
6	43% 46%	25% 30%	30% 40%	29% 42%	38% 35%	70% 90%	70% 88%
**Mean**	**48% 42%**	**22% 41%**	**35% 52%**	**32% 45%**	**36% 37%**	**76% 86%**	**75% 84%**

RAMP1 was expressed in the cytoplasm of large- and medium-sized neurons and in thick nerve fiber (presumably Aδ-fibers) (Fig. 14b).

In addition, the number of positive neurons was higher in females (41%) when compared to males (22%) (Table 3).

#### Distribution of OT and OTR in the trigeminal ganglion

**Fig. 15:**
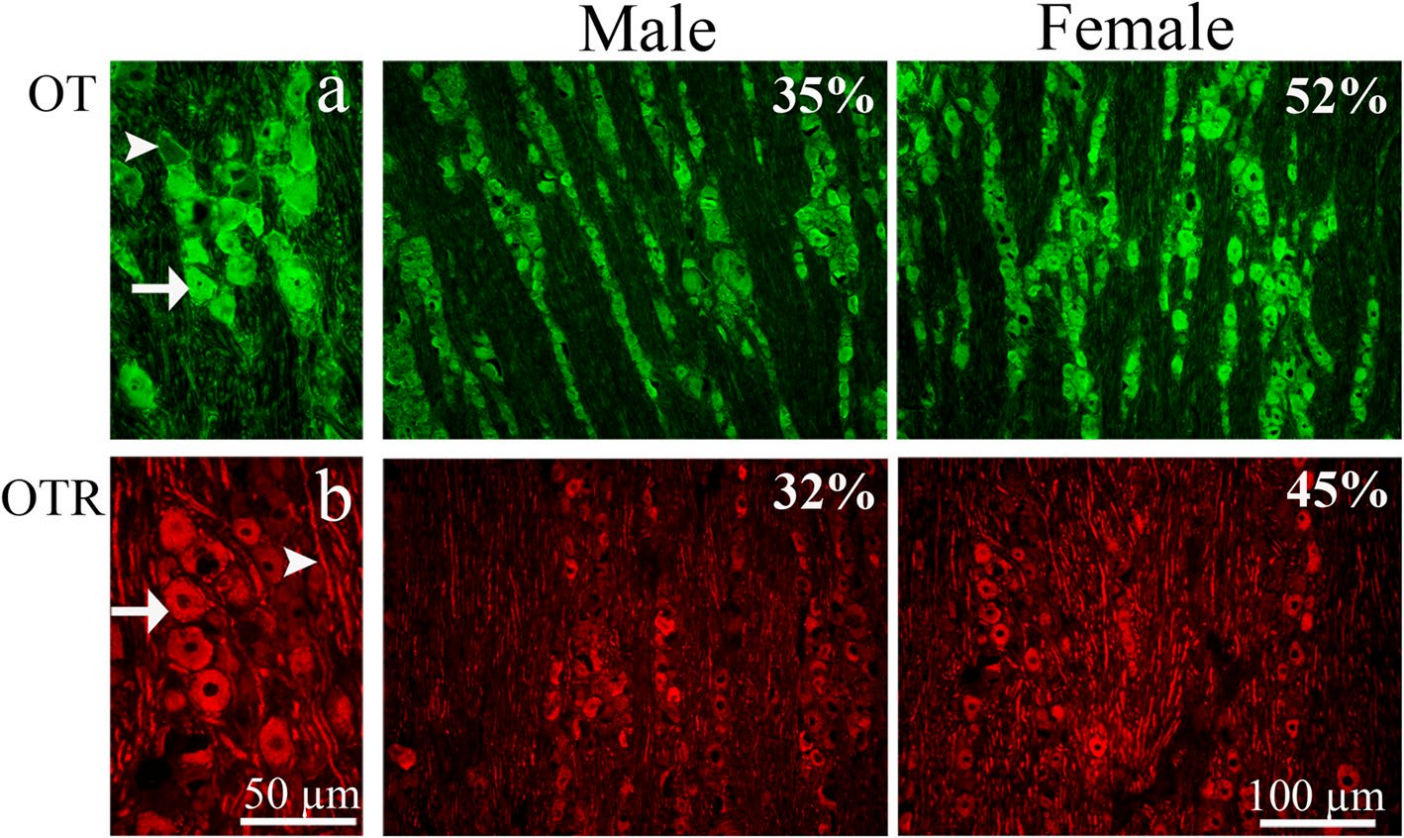
Immunohistochemistry of OT and OTR expression in TG from male and female rat. **a** OT immunoreactivity was located in the SGCs (arrowhead) and in the cytoplasm of neurons (arrow). Of all neurons, 35% in males and 52% in females showed OT immunoreactivity. **b** OTR (32% in males. 45% in females) was expressed in the neuronal cytoplasm (arrows) and in thick nerve fiber (Aδ –fibers) (arrowheads)

According to our previous study, OT immunoreactivity is found mostly in the satellite glial cells (SGC) and in some TG neurons in the young male rats [32]. In the present study, we observed that there are some differences in the OT protein expression between female and male rats. In the female rats, the OT protein was located in most cases in the SGCs, but also in the cytoplasm of some large and medium sized neurons. However, in male rats OT immunoreactivity was present in the cytoplasm of all types of TG neurons (large, medium, and small sized) (Fig. 15a). In addition, we found that the OT immunoreactivity in the neurons and fibers were more intense, and the number of positive cells was higher in the female (52%) rats compared to the male rats (35%) (Table 3).

We observed OTR immunoreactivity located in the cytoplasm of large and medium sized neurons. OTR immunoreactivity was also found in thick nerve fibers (Aδ-fibers) (Fig. 15b). Furthermore, we observed that the OTR immunoreactivity in the neurons and fibers were more intense, and the number of positive cells was higher in the female rats (45%) compared to the male rats (32%) (Fig. 15b and Table 3).

#### Distribution of AVP and its receptors in the trigeminal ganglion

**Fig. 16:**
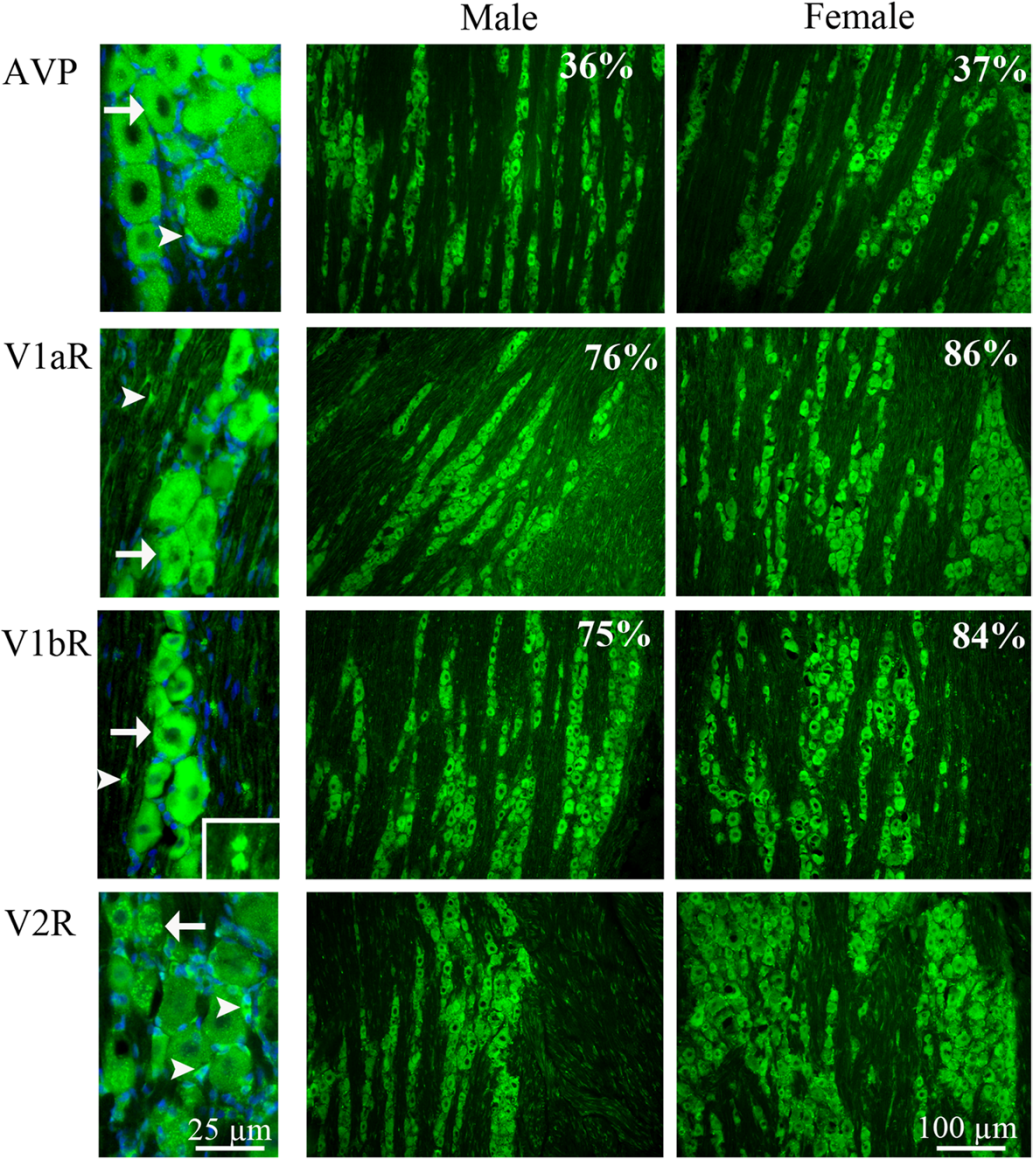
Immunohistochemistry of AVP, V1aR, V1bR and V2R in TG from male and female rat. AVP immunoreactivity was located in neuronal cytoplasm (arrow) and in SGCs (arrowhead) and of all neurons, 36% were positive for AVP in males and 37% in females. In males, 76% of all neurons were V1aR immunoreactive and 75% V1bR immunoreactive. In females, 86% of all neurons were positive for V1aR and 84% of all neurons were positive for V1bR. V1aR and V1bR were located in neuronal cytoplasm (arrow) and in fibers (arrowheads). V2R immunoreactivity was expressed mostly in the SGCs (arrowhead) and a fewer in the neuronal cytoplasm (arrow)

We observed AVP immunoreactivity located in the neuronal cytoplasm and in the SGCs. We counted cells and found that 36% of the total number of neurons expressed AVP in male rats and 37% in female rats (Fig. 16 and Table 3).

V1aR immunoreactivity was expressed in the cytoplasm of neurons, and we found that 86% of the total number of neurons was positive in female rats, which were higher compared to the male rats (76%) (Table 3). We also observed V1aR immunoreactivity in some of the SGCs, in the thin fibers (Fig. 16).

75% of all neurons expressed V1bR immunoreactivity in male rats and 84% in female rats. V1bR expression was located in the neuronal cytoplasm (Fig. 16 and Table 3). Overall, the AVP receptors expressions were very rich in the TG.

V2R immunoreactivity was located mostly in the SGCs, and only few in the cytoplasm of large and medium sized neurons. There were no differences in the V2R protein expression between the female and male rats (Fig. 16). Since V2R is mostly expressed in the SGC, no counting of V2R expressing neurons could be done.

#### Co-localization of AVP with OTR, and V1bR with CASPR

**Fig. 17:**
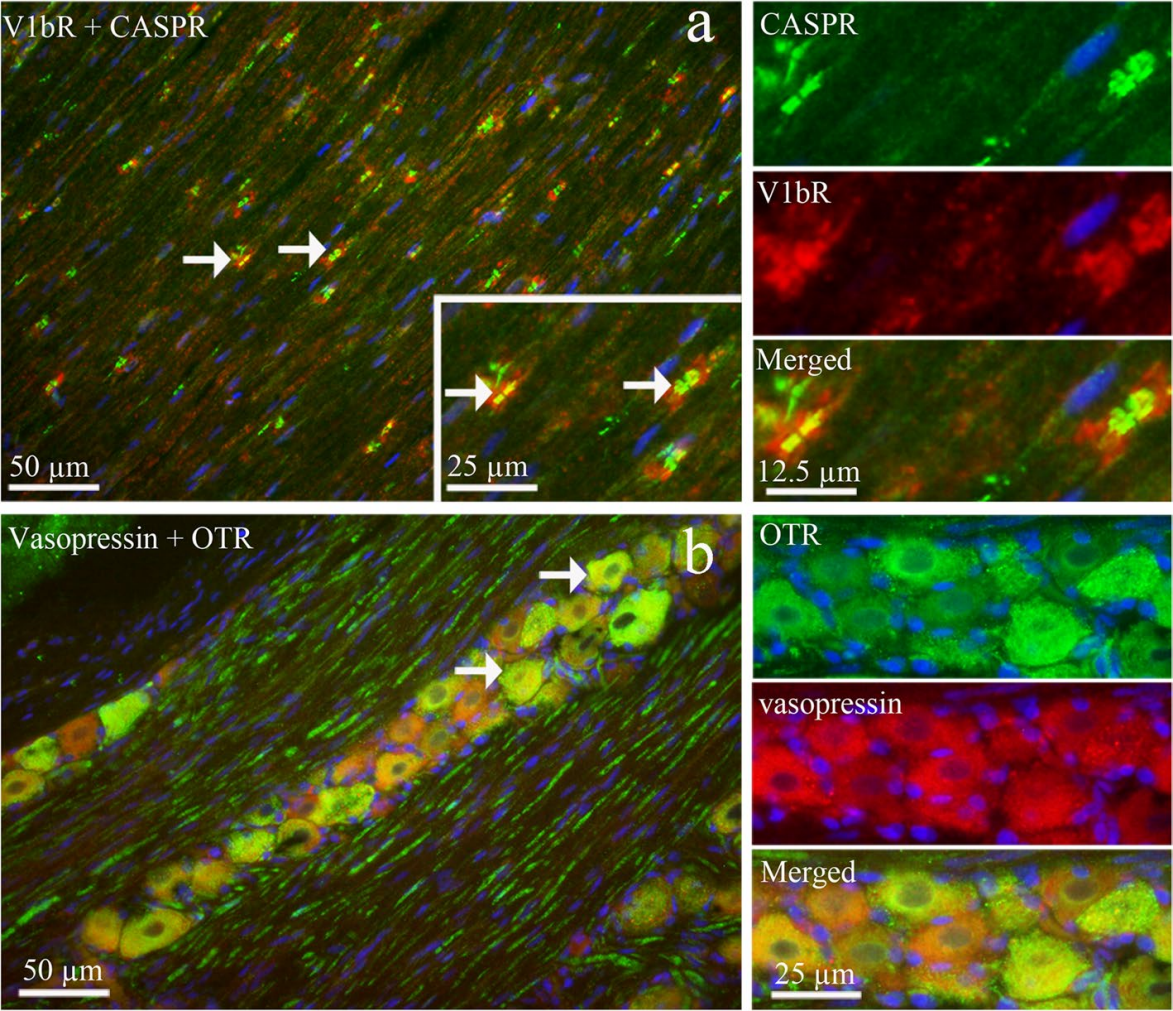
Co-localization of V1bR with CAPSR and vasopressin with OTR in TG**. a** Double staining of V1bR and CASPR showed that V1bR co-localized with CASPR in the node of Ranvier (arrows). **b** Double staining of vasopressin and OTR showed that vasopressin co- localized with OTR in the cytoplasm of some medium sized neurons (arrows)

Double immunohistochemistry was performed to investigate co-localization of V1bR and CASPR, which is a membrane protein and expressed at the nodes of Ranvier of myelinated fibers within the ganglion. We found that V1bR co-localized with CASPR in the nodes of Ranvier (Fig. 17a). The expression was specifically located to Aδ-fibers and indicated that V1bR expressed in the paranodal region of myelinated trigeminal axons. In addition, double staining of AVP and OTR showed co-localization in the cytoplasm in some TG neurons (Fig. 17b).

#### Co-localization of AVP, V1aR and V1bR with CGRP and RAMP1

**Fig. 18:**
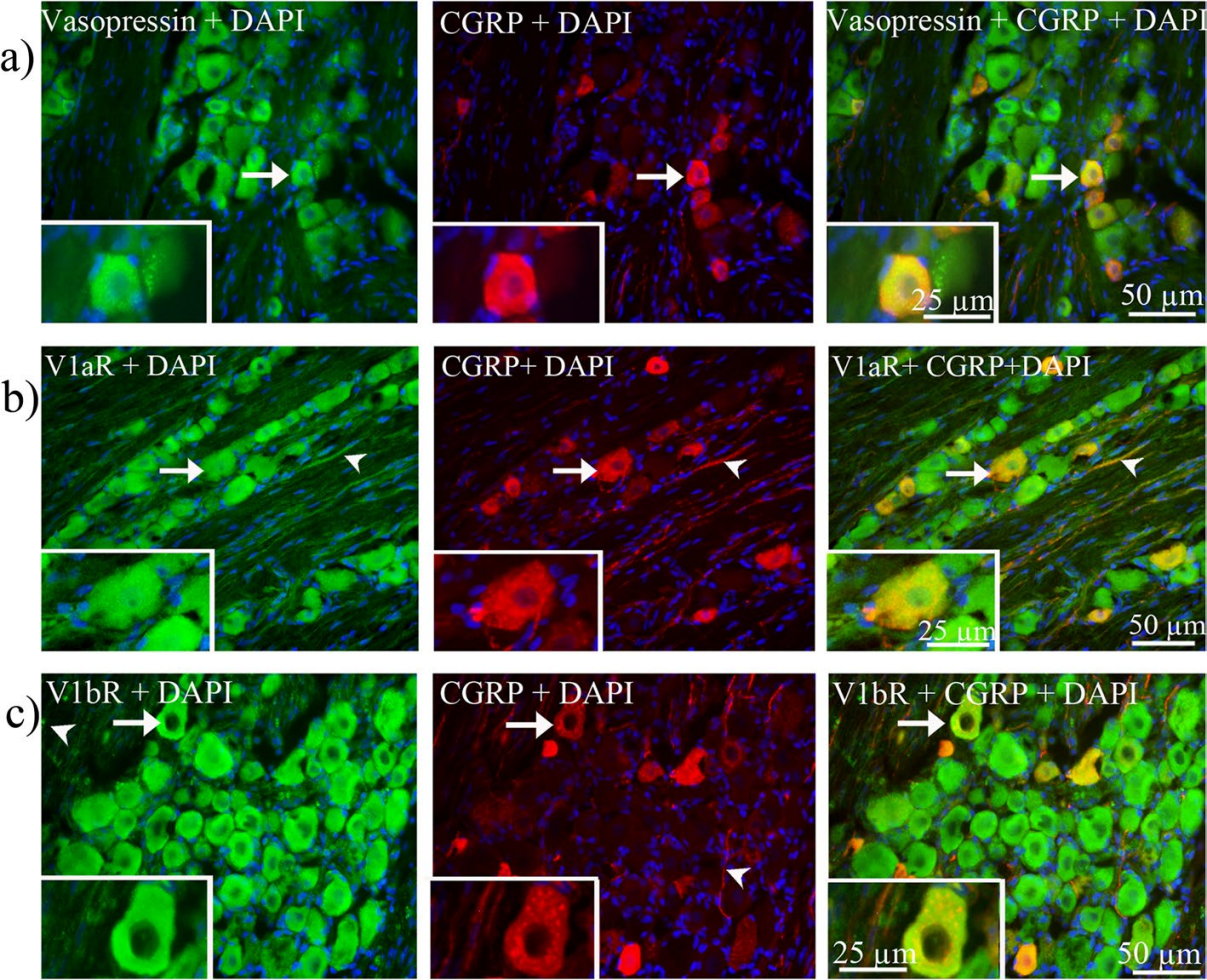
Co-localization of vasopressin and its receptors with CGRP in TG. **a** Double staining of vasopressin and CGRP **b** double staining of V1aR and CGRP, **c.** double staining of V1aR and CGRP. Vasopressin, V1aR and V1bR, co-localized with CGRP in the cytoplasm of TG neurons (arrows). V1aR and CGRP were co-expressed in a few fibers (arrowhead)

Double staining showed that AVP, V1aR and V1bR co-localized with CGRP in the neuronal cytoplasm of small and medium sized neurons in TG (Fig. 18a-c). Moreover, co-expression of V1aR and CGRP in a few fibers was observed (Fig. 18b).

**Fig. 19:**
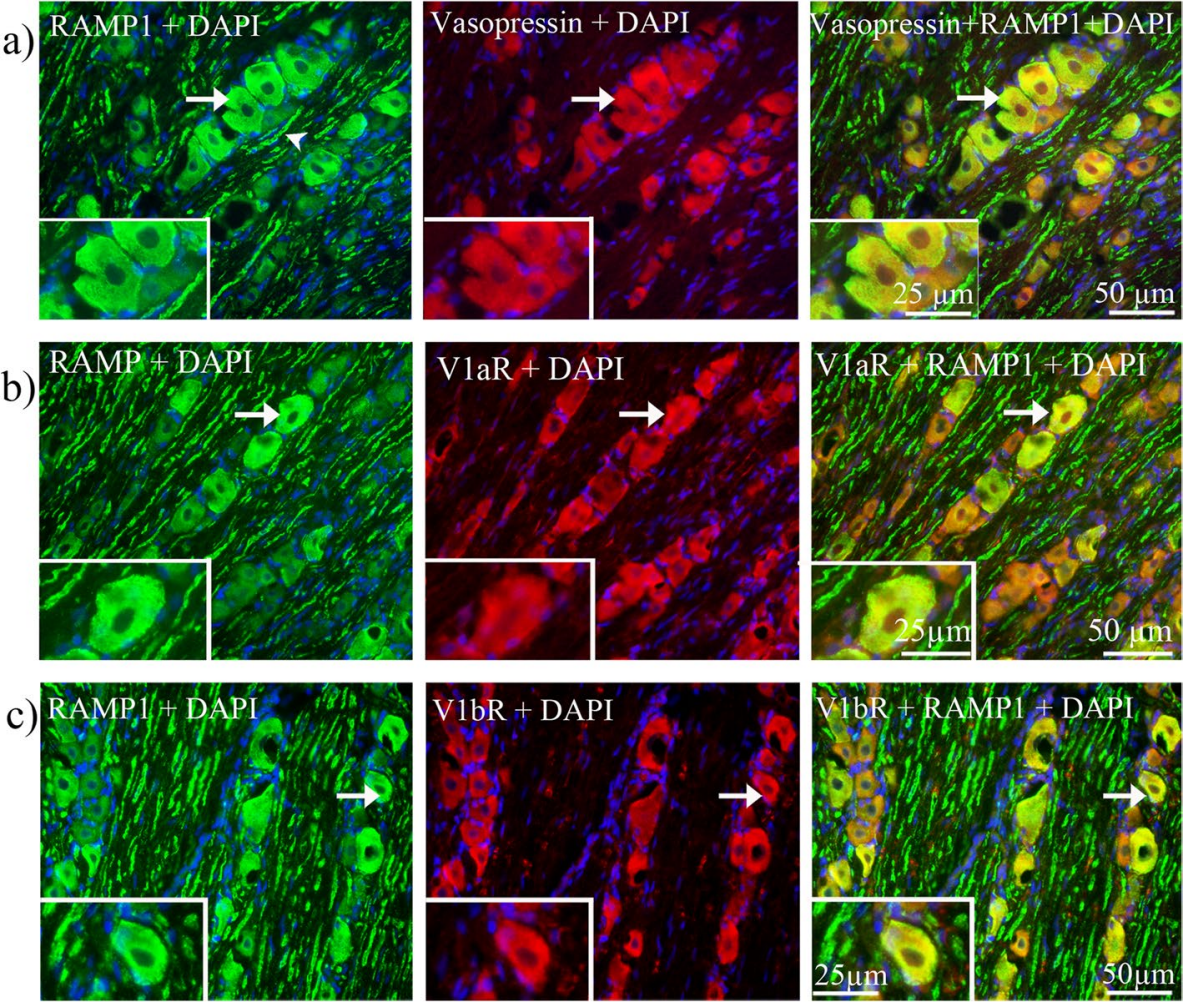
Co-localization of vasopressin and its receptors with RAMP1 in TG. **a** Double staining of vasopressin and RAMP1, **b** double staining of V1aR and RAMP1, **c** double staining of V1bR and RAMP1. Vasopressin, V1aR and V1bR co-localized with RAMP1 in the neuronal cytoplasm (arrows)

Next, we evaluated if AVP and its receptors co-localized with RAMP1. As shown in Fig. 19a-c, we found that AVP, V1aR and V1bR co-expressed with RAMP1 in the cytoplasm of large and medium sized neurons.

### The mRNA expression of AVP, V1aR and V1bR in hypothalamus and TG

**Fig. 20:**
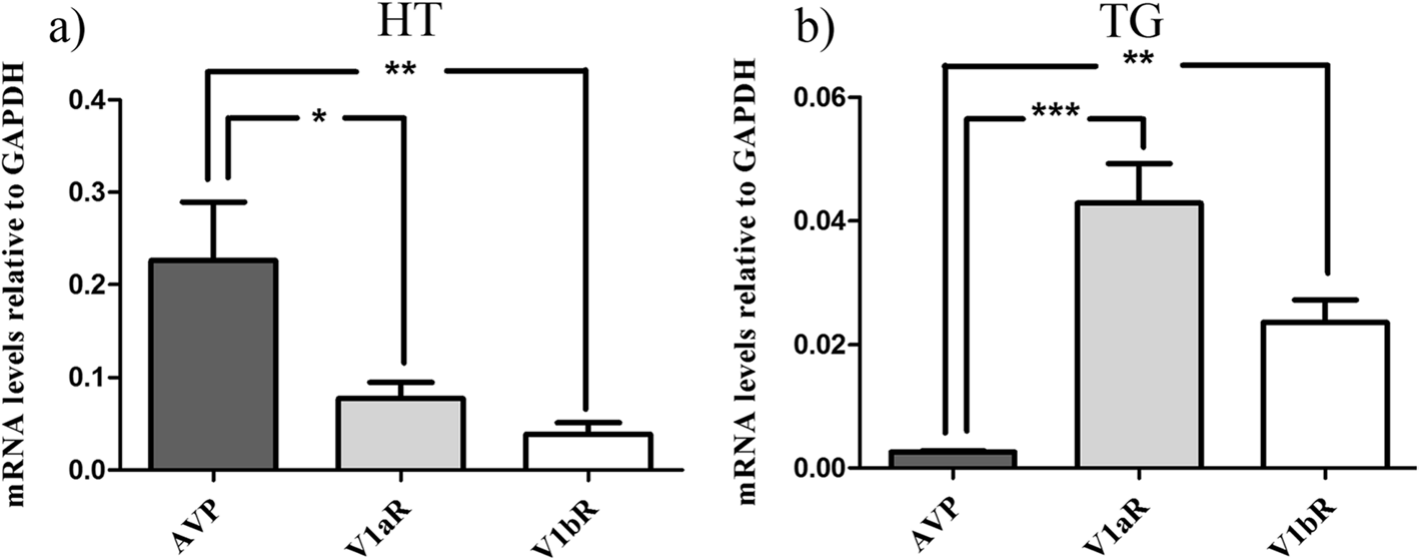
Expression of AVP, V1aR and V1bR mRNA in hypothalamus and trigeminal ganglion. **a** The mRNA levels of AVP, V1aR and V1bR in HT and, **b** in TG. Expression was normalized to housekeeping gene GAPDH. Data were obtained by real-time PCR and are expressed as mean ± s.e.m., *n* = 6, *P < 0.05, **P < 0.01 and ***P < 0.001

To determine whether the AVP, V1aR and V1bR genes are expressed in the hypothalamus and TG, real-time PCR analysis of these tissue mRNA was performed. In each qPCR experiment either a no temple control (water control) or a minus RT control was included, and there were no signs of contamination and/or genomic DNA in those samples. The mRNA expression of AVP, V1aR and V1bR were normalized to the house keeping gene GAPDH (Fig. 20). The experiments confirmed the AVP mRNA expression of the AVP (Ct: 18.46 ± 0.59), the V1aR (Ct: 19.98 ± 0.41) and the V1bR (Ct: 21.22 ± 1.14) in hypothalamus, and also the AVP (Ct: 24.12 ± 0.34), the V1aR (Ct: 20.21 ± 0.65) and the V1bR (Ct: 21.11 ± 0.77) in TG. Statistical analyses revealed significant differences between vasopressin and its receptors. The AVP mRNA was abundantly expressed in the hypothalamus, on the contrary to TG where AVP mRNA was expressed to a very low degree (Fig. 20). The V1aR mRNA was expressed higher than V1bR mRNA in both hypothalamus and TG (Fig. 20).

The values for mRNA levels of AVP, V1aR and V1bR in both HT and TG are shown in Additional file 1: supplementary Table 1.

## Discussion

Interestingly, in the 1980s and 1990s, the AVP system dominated scientific attention. It originally looked as if AVP is crucial for male allo-parental behaviour and partner preference, whereas OT is essential for female pair bonding [42, 43]. Immunohistochemical demonstration of AVP and OT containing pathways within the brain were described in the − 80 [44]. In addition, the concept that a neuron may release more than one active substance was expanding in CNS [45]. These findings lead to questions about the general role of the AVP and OT systems.

Migraine is a major neurological disorder that disables 17% of women. A better understanding of potential hormonal and genetic influences on migraine mechanisms is needed to specifically improve treatment of women suffering from this disease. We have earlier examined reproductive hormones e.g. estrogen and OT, in the trigeminovascular and central pain pathways related to migraine [30, 33]. In the present paper, we examined the distribution of the molecular relative to OT - AVP and its receptors - in CNS and TG. Since AVP is considered a stress hormone and is released in response to pain and stress, it therefore may have association with migraine pathophysiology.

### AVP and the brain

CGRP and its receptor are hot topics in migraine and has successfully resulted in several monoclonal antibodies towards CGRP and CGRP receptor, as well as a group of small CGRP receptor antagonistic drugs which are effective and show little adverse events [31, 46]. Our previous study showed that CGRP is present in almost all neuronal cell soma in the brain [47]. The receptor was almost exclusively found in the neuronal processes. Moreover, we examined the CNS distribution of OT/OTR with a focus on migraine-related regions and areas expressing CGRP/receptors [32]. In many regions, in particular regions harbouring the “migraine generator” in the brainstem, we found correlation between the three types of mappings. We proposed that a central role for OT will be a key to understanding more about migraine pathophysiology, in particular migraine pathophysiology of females. The role of OT/OTR in modifying the CGRP system is still enigmatic, but their differential expression suggest involvement both of central and trigeminal OT pathways which may modify each other, centrally on the initiation of an attack and peripherally on the headache phase.

In addition to the OT distribution, we mapped estrogen receptors in CNS and TVS. Clearly, there are numerous expression sites for estrogen receptors both in CNS and in the TVS, all of these sites share a relation to migraine pathophysiology [33]. We further showed that the female middle cerebral artery response to ERβ in a stronger fashion than that for the male counterpart while the ERα responses were similar. Together, this points to a hypothesis where estrogen could have a modulatory role on the trigeminal neuron function in general, as well as on intracranial vasodilation, rather than on the acute CGRP release mechanisms.

In the present study we confirmed results from several other studies showing that the two clusters of cell bodies in the hypothalamus - PVN and SON - contain the important neuropeptide AVP, a molecular relative to OT [48]. Comparing the distribution of AVP with the “migraine generator” and CGRP, OT/OTR and estrogen receptors, showed that almost all of the peptides (including AVP) were found in migraine-related areas such as cortex, cerebellum, hypothalamus, amygdala and pons (Fig. 1a). The degree of expression of AVP and AVP receptors, especially V1bR, in the CNS is complex and suggestive of many different brain functions, which does not exclude a possible role in migraine. Interestingly, we found AVP expression, however weaker compared to other brain areas, in Sp5 (spinal trigeminal nucleus caudalis; TNC), which constitutes an important part of the pain pathways activity in migraine attacks.

In the present study, we have showed results from male rat brains, mainly because of the possibility to be able to compare our results with previous results on CGRP and OT, their receptors and estrogen receptors. However, we have some results from female rat brains: three female rats were used, and these showed the same pattern of immunoreactivity of the different brain regions as for the male rat brains. However, the female brains showed in general more intense immunoreactivity, especially for the V1bR antibody. In addition, more cortical areas displayed immunoreactivity in the females and more neurons in the different nuclei of the brainstem and in red nucleus (R) expressed AVP and receptors. The findings only include three brains and, therefore, further validation must be performed to find out whether there exists a significant difference between male and female brains.

By using qPCR we showed that AVP, V1aR and V1bR were expressed in the hypothalamus, which is in agreement with our present immunohistochemistry results and with evidence from previous studies [49, 50]. In those studies, Hallbeck and Hurbin used in situ hybridization and real-time PCR methods and reported AVP, V1aR and V1bR mRNA expression in CNS and particularly the hypothalamus in rat. Thus, in light of the key role of initiation and maintenance of migraine attacks by the hypothalamus, it is particularly fascinating that AVP is an output that influences trigeminal, spinal and central brain areas associated with migraine.

Several studies indicated the role of hypothalamus in migraine [51, 52] by involving in the processing of trigeminal nociceptive signaling, which is processed in TG and sent to the CNS via projections of the trigeminal neurons that terminate in the trigeminocervical complex (trigeminal nucleus caudalis; Sp5, and the upper levels of the spinal cord) [53, 54], that results in the pain phase of migraine. During stress, the PVN and SON of the hypothalamus release hormones including OT, AVP and corticotrophin–releasing hormone [55]. Moreover, observations point to a strong correlation between neurohormones and migraine attacks in women [5].

### AVP and TG

In the present study, we have focused on migraine-related structures, including the hypothalamus and other central areas as well as the TVS pathway. In these CNS structures, TG is a key component for understanding male-female differences and hormonal influences in migraine [31, 56].

In a previous study, Kai-Kai and associates [57] reported the immunoreactivity of OT and AVP in male rat TG. In the present study we showed similar results in both mRNA and protein levels. However, only low mRNA level of AVP was detected. By using immunohistochemistry, we showed that AVP was expressed in the neuronal cytoplasm and in the SGCs in both male and female rats. Importantly, AVP function is intrinsically dependent on its receptors; therefore, to examine this, we investigate AVP receptors expression in TG. We found high expression of V1aR and V1bR proteins in TG; the expression was mainly found in the cytoplasm of TG neurons and in some SGCs, whereas V2R immunoreactivity was seen predominantly in SGCs. The present study identified the expression of AVP, V1aR and V1bR in both mRNA and protein level in the TG. Furthermore, it is the first study that showed the AVP and V1aR and V1bR immunoreactivity co-localized with CGRP, RAMP1 and OTR positive cells, and the amount of V1aR and V1bR immunoreactivity cells were higher in the TG of female rats. The different types of fibers are involved in the different aspects of pain perception. In the trigeminal ganglion, CGRP is expressed in C-fibers and its receptor is expressed in Aδ-fibers [58]. C-fibers are un-myelinated fibers that project to the lamina I and II in the trigeminal complex of the brainstem. These fibers respond to stimuli which have strong intensity and are responsible for the lasting and slow sensation of pain, termed as second pain. Aδ-fibers are myelin-ensheathed fibers that project to the lamina III and IV. These fibers respond to a weaker intensity of stimuli and are responsible for the sensation of a quick shallow that is specific on one area and termed as first pain [54, 59, 60]. Interestingly, we found that the V1aR co-localized with CGRP in C-fibers (second pain) (Fig. 17b) and V1bR co-localized with CASPR in Ranvier nodes in the Aδ-fibers (first pain) (Fig. 16a). Our results may suggest a functional role of AVP receptors in both C-fibers and Aδ-fibres, which may relate to migraine pathophysiology.

Previously, we have demonstrated the expression of CGRP, RAMP1, OT and OTR in the TG from male rat [61, 62]. Here, we expanded this and examined if sex showed any differences in these protein expressions. We found no differences in the expression of CGRP and AVP immunoreactive cells between male and female rats. On the other hand, the expression of the RAMP1, OT, OTR, V1aR and V1bR immunoreactive cells were more intense and the numbers of positive neurons were higher in female compared to male rats. We have no explanation for this difference but encourage future functional studies.

TG is a key focus for understanding male-female differences and hormonal influences in migraine. OT can suppress migraine attacks [63]. TG expresses OTR mRNA and protein [32, 64], indicating direct regulation by OT. Overall, receptor mapping studies reveal strong evidence for estrogen and OT regulation of CGRP rich migraine-related regions. AVP is a molecular relative to OT. Our results show that AVP is similarly expressed in the brain and TG, as for other neurohormones. However, there was no significant difference between the sexes in the number of cells that expressed AVP. But the number of vasopressin receptors (V1aR and V1bR) immunoreactivity was higher and more intense in female rats.

## Conclusion

In conclusion, we showed that AVP and its receptors were expressed in migraine-related areas within the male brain, and in female and male TG, predominantly in neuronal cytoplasm. Also, we observed that there were differences in the number of positive V1aR, V1bR, RAMP1, OT and OTR immunoreactive cells between male and female rats in TG. Furthermore, AVP and its receptors mRNA were expressed in both hypothalamus and TG. Currently there is vast interest in migraine treatment following the successful introduction of CGRP comprised drugs. At the same time, a better understanding of potential hormonal influences on migraine mechanisms is needed to improve treatment of female migraineurs. It is intriguing that AVP is an output of hypothalamic neurons that influences trigeminal, spinal and central brain areas associated with migraine. Therefore, AVP and its receptors might be components that contribute to the hormonal influence on migraine in women.

## Supplementary Information

**Additional file 1: Supplementary Table 1.** mRNA values for AVP, V1aR and V1bR in HT and TG.

## Data Availability

The datasets generated during and/or analyzed during the current study are available from the corresponding author on reasonable request.

## Abbreviations

AH: Anterior hypothalamic area
AVP: Arginine vasopressin
BSA: Bovine serum albumin
CA1: Field CA1 of the hippocampus
CA2: Field CA2 of the hippocampus
CA3: Field CA3 of the hippocampus
CASPR: Contactin Associated Protein 1
Cb: Cerebellum
cc: Corpus callosum
cDNA: Complementary DNA
CGRP: Calcitonin gene-related peptide
CNS: Central nervous system
DAPI: 4′, 6-diamidino-2-phenylindole
dhc: Dorsal hippocampal commissure
DNA: Deoxiribonukleinsyra
ER α and β: Estrogen receptors α and β
Gi: Gigantocellular reticular nucleus
GPER: G protein-coupled estrogen receptor 1
Gran layer: Cerebellar granular layer
GAPDH: Glyceraldehyde-3-phosphate dehydrogenase
HT: Hypothalamus
ICj: Islands of Calleja
IO: Inferior olive
LC: Locus coeruleus
LPB: Lateral parabrachial nucleus
Me5: Mesencephalic trigeminal nucleus
Med: Medial cerebellar nu
Mol layer: Cerebellar molecular layer
mRNA: Messenger RNA
MVPO: Medioventral periolivary nucleus
OT: Oxytocin
OTR: Oxytocin receptor
ox: Optic chiasm
PBS: Phosphate buffer saline
PBS-T: Phosphate buffer saline-TritonX
Pc: Purkinje cell
PF: Paraformaldehyde
Pn: Pontine nuclei
PnO: Pontine reticular nucleus
PVA: Paraventricular thalamic nu
PVN: Paraventricular hypothalamic nu
R: Red nucleus
RAMP1: Receptor activity-modifying protein 1
RNA: Ribonucleic acid
Rt-qPCR: Real-time quantitative Polymerase Chain Reaction
SGC: Satellite glial cell
SHy: Septohypothalamic nuleus
SON: Supraoptic nucleus
Sp5: Spinal trigeminal nu
tfp: Transverse fibers pons
TG: Trigeminal ganglion
TVS: Trigeminovascular system
tz: Trapezoid body
Tz: Nucleus of trapezoid body
V1aR: Vasopressin receptor 1A
V1bR: Vasopressin receptor 1B
V2R: Vasopressin receptor 2
vhc: Ventral hippocampal commissure
