# Supplementary material for: Expression of vasopressin and its receptors in migraine-related regions in CNS and the trigeminal system: influence of sex
Source: J Headache Pain. 2022 Dec 1;23(1):152. doi: 10.1186/s10194-022-01524-7 (PMC9713967; doi:10.1186/s10194-022-01524-7)
Supplement: Supplementary file 1 — Additional file 1: Supplementary Table 1. mRNA values for AVP, V1aR and V1bR in HT and TG. [file 10194_2022_1524_MOESM1_ESM.docx]

**[Additional file 1: Supplementary Table 1](https://static-content.springer.com/esm/art%3A10.1186%2Fs10194-022-01394-z/MediaObjects/10194_2022_1394_MOESM3_ESM.docx). mRNA values for AVP, V1aR and V1bR in HT and TG.**

| **mRNA values** | | | | | | |
| --- | --- | --- | --- | --- | --- | --- |
|  | AVP | | V1aR | | V1bR | |
|  | HT | TG | HT | TG | HT | TG |
| Mean | 0,2263 | 0,002599 | 0,07750 | 0,04281 | 0,03870 | 0,02357 |
| S.D | 0,1546 | 0,0005077 | 0,04224 | 0,02021 | 0,03022 | 0,01161 |
| S.E.M | 0,06310 | 0,0001606 | 0,01724 | 0,006390 | 0,01234 | 0,003672 |

Data were obtained by qPCR and values were expressed relative to GAPDH levels for 6 rats per group.

Abbreviations; mRNA: messenger RNA, AVP: Arginine vasopressin, V1aR: Vasopressin receptor 1A, V1bR: Vasopressin receptor 1B, HT: Hypothalamus, TG: Trigeminal ganglion, qPCR: quantitative Polymerase Chain Reaction, GADH: glyceraldehyde-3-phosphate dehydrogenase.
